# Interpersonal motor synchrony in autism: a systematic review and meta-analysis

**DOI:** 10.3389/fpsyt.2024.1355068

**Published:** 2024-02-19

**Authors:** Laura Carnevali, Irene Valori, Giorgia Mason, Gianmarco Altoè, Teresa Farroni

**Affiliations:** ^1^ Department of Developmental Psychology and Socialization, University of Padova, Padova, Italy; ^2^ Chair of Acoustics and Haptics, Technische Universität Dresden, Dresden, Germany; ^3^ Centre for Tactile Internet with Human-in-the-Loop (CeTI), Technische Universität Dresden, Dresden, Germany

**Keywords:** interpersonal motor synchrony, autism spectrum disorder, social interactions, neurodiversity, individual differences

## Abstract

**Introduction:**

Interpersonal motor synchrony (IMS) is the spontaneous, voluntary, or instructed coordination of movements between interacting partners. Throughout the life cycle, it shapes social exchanges and interplays with intra- and inter-individual characteristics that may diverge in Autism Spectrum Disorder (ASD). Here we perform a systematic review and meta-analysis to summarize the extant literature and quantify the evidence about reduced IMS in dyads including at least one participant with a diagnosis of ASD.

**Methods:**

Empirical evidence from sixteen experimental studies was systematically reviewed, encompassing spontaneous and instructed paradigms as well as a paucity of measures used to assess IMS. Of these, thirteen studies (n = 512 dyads) contributed measures of IMS with an in situ neurotypical partner (TD) for ASD and control groups, which could be used for meta-analyses.

**Results:**

Reduced synchronization in ASD-TD dyads emerged from both the systematic review and meta-analyses, although both small and large effect sizes (i.e., Hedge’s g) in favor of the control group are consistent with the data (Hedge’s g = .85, p < 0.001, 95% CI[.35, 1.35], 95% PI[-.89, 2.60]).

**Discussion:**

Uncertainty is discussed relative to the type of task, measures, and age range considered in each study. We further discuss that sharing similar experiences of the world might help to synchronize with one another. Future studies should not only assess whether reduced IMS is consistently observed in ASD-TD dyads and how this shapes social exchanges, but also explore whether and how ASD-ASD dyads synchronize during interpersonal exchanges.

## 1 Introduction

Interpersonal synchrony (IS) is defined as the spontaneous rhythmic and temporal coordination of actions, emotions, thoughts, physiological, and neural processes between two or more people (1, 2). As depicted in this definition, the concept of interpersonal synchrony encompasses behavioral, physiological, and neural levels of analysis of the dynamics occurring between interacting individuals. In recent years, several authors have attempted to embed recent findings in a more comprehensive context ultimately providing a broader definition of the terms themselves [for review, (2–4)]. Focusing on the motor aspects of IS, hereafter referred to as Interpersonal Motor Synchrony (IMS), evidence shows that people interact through coordination of eye and body movements, posture, gestures, and facial expressions across the entire life cycle (5, 6). An important distinction has to be made between behavioral matching and interpersonal motor synchrony, both of which are considered part of a broader construct, namely interpersonal coordination. Behavioral matching, often referred to as behavioral mimicry, occurs when individuals mirror each other’s behavior within a brief timeframe (7). Unlike behavioral mimicry, IMS refers to a continuous and complex coordinated sequence of actions that two or more individuals perform together in a given time stream, encompassing not only identical behaviors but also complementary actions (2, 8). Movements can be at an equivalent (in-phase) or at opposite (anti-phase) point of the cycle at a given time (9). As such, IMS is only partially superimposable on mimicry and imitation, which involve the exact reproduction of other’s action and can occur in deferred time with respect to the action to be imitated (9). Further distinction refers to the spontaneous, instructed, or induced nature of IMS. ‘Spontaneous’ refers to unintentional motor alignment (i.e., spontaneously rocking at the same pace, adopting the same posture etc.), ‘instructed’ implies being given specific instructions (i.e., step, tap finger, press a button with the partner), while ‘induced’ implies passively receiving stimulation from a third party such that the participants’ movements are aligned [i.e., experimenters rocking children on a swing at the same pace or bouncing at the same rhythm such that infants they are holding also bounce synchronously; see for example (10, 11].

Crucially, the mechanisms that allow individuals to synchronize during live interpersonal exchanges pertain to a growing research field (12, 13). Both low-level processes and higher-level cognitive functions contribute to the way individuals structure their interactions. Some common bases in low-level sensory, motor, and predictive processes might help us spontaneously synchronize, while a particular involvement of higher-level cognitive processes may additionally support voluntary synchronization. However, we do not know much about what neuropsychological bases and effects on social exchanges differentiate spontaneous and instructed synchrony, which are constantly intertwined in daily social intercourses.

### 1.1 Interpersonal motor synchrony: developmental foundations and relevance

Interpersonal synchrony is fundamental in early social development, with cascading effects on sensory, motor, and cognitive functions. Hoehl et al. (13) emphasize that synchronization hinges upon the intertwin of lower- and higher-level factors. The first include grasping temporal regularities and interaction-specific rhythm, while the second encompass the attribution of value to oscillatory stimuli, along socio-emotional elements like the nature of the relationship and one’s associated emotions. Additional elements identified as crucial for the synchronization process include social orientation, selective attention to social cues, multisensory processing, action prediction, motor behavior planning and execution, as well as monitoring and adaptation (14). Exploring these facets across development and critical windows is essential to grasp potential deviations in their trajectories.

From the last trimester of gestation, biological rhythms provide a neuro-biological substrate for coordinated interaction, which blossoms at birth, with infants being able to detect behavioral contingency and parents spontaneously responding to their infants’ cues in a contingent fashion (15–17). Across the first year of life, infants experience multimodal synchrony through vocalization, facial expressions, affective touch, body proximity, and movements, they develop intentionality and become active agents of synchrony (16). In addition to the integration of contingent multisensory information, IMS arises from the sensitivity to temporal contingency between self and others’ movements, which is related to the sense of social agency (18). Indeed, being able to detect the timing and the temporal contingency of social events is associated with higher predictive abilities and increased interpersonal synchronization (19).

Crucially, acting in synchrony with interactive partners increases social connection and improves communication exchanges (20). Numerous studies observed a positive association between joint motor action and social bonding tendency [*for a review*, see (6)]. Indeed, beyond the different definitions and distinctions (e.g., motor, physiological, neural; spontaneous, instructed), IS increases prosocial behaviors and attitudes, perceived social bonding, and social cognition, thus playing a relevant role in the development of self-regulation, symbolic, and predictive abilities (2). Specifically, IMS appears to have a beneficial impact on prosociality and social development from early in life (21). For instance, it has been shown that synchronous (vs asynchronous) movements enhance children’s dyadic cooperation (10) and increase the frequency of prosocial behaviors (22). Across the lifespan, IMS fosters interpersonal cooperation, sharing, helping and trust, affiliation, feeling of closeness, and empathy (23–26). Moreover, IMS affects individuals’ sensory thresholds and positively affects intrapersonal mechanisms such as self-evaluation and sense of agency (26). One hypothesis concerning the mechanism by which synchronizing with the other promotes social attitudes and prosocial behaviors in different age groups (21, 27, 28) relates to a self-other overlap. More specifically, it is thought that continuous overlap between self- and other-generated movements contributes to feelings of increased similarity and closeness, as well as to better prediction of others’ actions (13). Such modulation of psychological boundaries between self and other could foster cooperation (29) and more generally increase social cohesion (30, 31). In addition, IMS can increase prosocial behaviors by facilitating emotional contagion and enhancing perceptual and attentional biases towards synchronous counterparts (23). IMS is in fact suggested to scaffold real-time tracking of other’s feelings (32), which promotes emotional contagion and leads to increased empathy and as well as greater inclination to communicate and connect with others. Moreover, individuals manifest enhanced perceptual and attentional biases towards synchronous partners, leading to better memory for other-related information (33) and improved recognition of their faces (34).

In summary, the mechanisms underlying the positive consequences of IMS relate to self-other overlap, emotional contagion, and enhanced attention towards the other, all of which contribute to feelings of closeness and prosocial attitudes. Many of the processes that have been described as underlying, associated with, or facilitated by IMS become specialized and redefined over the lifespan, drawing individuals’ developmental trajectories. If IMS serves as a social glue (6), it can be hypothesized that difficulties in socio-communicative abilities might be accompanied and perhaps partly accounted for atypical interpersonal motor synchrony. In line, it has been suggested that interpersonal synchrony emerges differently, and is potentially reduced, in people with Autism Spectrum Disorder (ASD) (3), which is characterized by socio-communicative difficulties, and often accompanied by atypical sensory, motor, and cognitive functioning (35).

### 1.2 Interpersonal motor synchrony in autism spectrum disorder

The literature has privileged studying the processes that typically contribute to people’s ability to synchronize with others, thereby examining the neurodiversity within the autism spectrum. Evidence suggests that people with ASD manifest reduced spontaneous synchronization and impaired ability to voluntarily coordinate with neurotypical partners, as captured by several behavioral, physiological, and neural indices (3). However, IS is at least a dyadic process in which the two partners coordinate mutually, meaning that each of them brings their individual characteristics into the exchange. At the individual level, the *intra-personal* perspective considers the individual functional profile [e.g. sensory perception, self-cognition, self-regulation, emotional experience, learning, memory; (26)] as a drive for one’s ability to synchronize with others. These characteristics are often similar within one population, such as within the autism spectrum, and contribute to shared experiences of the internal and external world within a given neurotype. However, the emergence of synchrony in an interaction between two individuals is not solely influenced by their individual traits, but also by how these align at the inter-individual level, in other words by the extent to which individual characteristics of the interacting partners align and impact the interaction (3, 36–38).

Multiple recent theories converge in the idea that intra-personal and inter-personal characteristics are contributing to social interactions, by recognizing and emphasizing that, even in neurotypical functioning, the same person may synchronize more with one interacting partner and less with another. According to the Extended Model of Alignment proposed by Shamay-Tsoory et al. (39), there are three levels at which social alignment occurs: motor synchrony (i.e., structure, direction, and rhythm of movement), cognitive synchrony (i.e., thoughts, beliefs, perceptions, intentions, and attitudes), and emotional contagion (i.e., emotional states and expressions). These three levels influence each other, but in turn they are influenced by other social factors. For example, we synchronize more with people to whom we feel close, whom we like and who belong to our ingroup. This happens because of what is called a resource optimization principle according to which being in synchrony conserves computational resources and promotes the alignment and overlap of representations of self and other (40). How this synchronization occurs finds reason in the predictive processing framework, according to which the Bayesian brain computes and maintains probabilities of events in the environment or related to the self by combining previous experiences with new incoming sensory information. A continuous encoding of sensory information related to the self and the other occurs in the interaction, which is compared to the prior one has about the synchronous interaction itself. If a gap is detected between the prior and the sensory evidence, this will be considered a prediction error and a system aimed at reducing this gap will be set in motion. When the gap is no longer detected, a reward system is activated with cascading effects on the quality and outcome of the interaction itself (39). This model also points that motor synchrony positively correlates with feelings of closeness (28, 41) and that shared emotions enhance closeness (42), indicating a bidirectional relationship where alignment, likability, and closeness mutually influence each other as core mechanisms of connectedness.

The *inter-personal* perspective in IMS leads to wonder whether ASD-TD dyads are more heterogeneous than TD-TD or ASD-ASD dyads, and that dyads of autistic persons might share more similar sensorimotor profiles and social skills, resulting in easier synchronization. One could hence propose that there is no “unique way of synchronizing” but rather functional characteristics that make synchronization easier when shared between the two partners. This is in line with research on the “dual empathy problem” (36, 43) and the “dialectical misattunement hypothesis” (38). The first points out that the more different the attitudes and social orientations of interacting individuals are, the more one will struggle to understand the other, with their own communication and perception styles (36, 43) and the second posits that different predictive and interactional styles, rather than deficient neurocognitive functions, contribute to communicative mismatches and weak interpersonal coupling. This perspective also highlights the cumulative nature of mismatch, which can lead to impoverished opportunities to learn socially mediated knowledge and skills. Factors such as familiarity, similarity of interests and ways of processing the environment, and attitudes contribute to synchrony. Recent evidence indeed shows that IMS is modulated by contextual and personal factors (5) and that familiarity also modulates synchrony at the neural level (44). In addition, Bolis et al. (45) show that greater interpersonal similarity of autistic traits is associated with higher measures of closeness, acceptance, and help, independent of factors such as length of friendship, age, gender, and average level of autistic traits in the dyad (45). In natural contexts (e.g., inclusive schools), the social experience accumulated over time could lead children to interact in a privileged way with peers with a similar neurotype (46). Unfortunately, the IMS literature considering ASD-ASD dyads is extremely scarce. In the present work we will systematically review evidence derived from literature exploring how autistic intra-individual characteristics may contribute to reduced IMS (comparing ASD-TD with TD-TD dyads). Then, we make a step further discussing the available evidence to scout the inter-personal perspective within the autistic population (ASD-ASD).

To investigate intra-personal precursors of synchrony that might contribute to diversity in developmental trajectories, some work has been done on siblings of ASD individuals, who are more likely to be diagnosed with ASD (47). These children showed lower synchrony during free play at 4 months of age, specifically when interactions were led by the infant (48). Evidence from neurotypical populations with autistic traits suggested a possible relation between IS and communication skills (49–51). For the scope of our work, we solely focused on IMS in both children and adults with ASD, excluding studies on siblings to reduce heterogeneity. We acknowledge that some studies investigated IMS toward digital stimuli (e.g., videos) or semi-human partners (e.g., robots or animations, virtual agents and avatars) of people with ASD (52–56). However, the type of stimulus one has to align with matters, as people have different perceptual and interactive skills with others in real or virtual situations (57, 58). We have therefore delved into naturalistic in-person interactions with human partners, to study this phenomenon in situations as similar as possible to those in daily life.

Building on this theoretical synthesis on the relevance of motor synchrony in optimizing social interactions, the present paper focuses on the extent to which dyads where at least one of the members is within the autism spectrum are able to synchronize from a motor perspective, in contrast to neurotypical dyads. Here, we do not focus on the outcomes of engaging in IMS, such as social bonding, quality of interaction, and rapport. Neither do we delve into the mechanisms by which these outcomes are achieved, such as self-other overlap, emotional contagion, and attention to the other. Instead, our hypothesis revolves around the idea that difficulties in social-communicative skills, frequently encountered by individuals on the autism spectrum, might be accompanied by atypical IMS. Therefore, in the present work we first present a systematic review to summarize the extant literature on IMS in dyads including at least one participant with a diagnosis of ASD, then we perform a meta-analysis to provide a quantification of the effect size regarding the phenomena of interest.

## 2 Methods

We followed the Preferred Reporting Items for Systematic Reviews and Meta-Analyses (PRISMA) guidelines for conducting and reporting meta-analysis and systematic reviews (59).

### 2.1 Literature search

Study searches were conducted up to November 14th, 2023 by means of EBSCO (hosting APA PsycArticles, APA PsycInfo), Elsevier’s Scopus®, and Pubmed® databases to select papers on the topic of IMS in autism. We used the following string: {[(sync* OR coordination OR entrainment) AND interpersonal AND (motor OR motion) AND (autis* OR asd OR asc)].ti,ab,kw.}. Our searches yielded to *n* = 515 manuscripts in total, selected from areas of Psychology, Neuroscience, and Social Sciences as published or in press articles written in English; then, duplicates were removed resulting in *n* = 445 papers to be screened. Two independent researchers (LC and IV) screened all the records by title and abstract, then read all the selected papers. A systematic coding form was employed to record specific reasons for exclusion at each stage of the selection process. After independent coding, disagreements in the assessment were resolved through pairwise discussions until consensus was reached. During title and abstract screening *n* = 379 papers were excluded for the following reasons: reviews or meta-analyses (*n* = 74), absence of ASD or control group (*n* = 247), out of topic (*n* = 58). An additional *n* = 3 papers identified via citation searching were added. After full-text reading (*n* = 69), further *n* = 53 papers were excluded, such that *n* = 16 papers were considered eligible for systematic review. Of these, *n* = 2 had to be excluded from the meta-analysis as the authors did not share the data, resulting in *n* = 14 papers available for analyses to this point.

### 2.2 Eligibility criteria

Eligible studies were written in English (selected at the database search phase), quantitative, and published in peer-reviewed scientific journals. To be included in the systematic review and meta-analysis, studies had to employ experimental designs and kinematic measures to investigate IMS between participants with and without ASD. More specific inclusion and exclusion criteria adopted for study selection are reported in **Table 1**.

**Table 1:** Eligibility criteria.

	Inclusion criteria	Exclusion criteria
*Language*	Written in English	Other than English
*Source and type of publication*	Research article published in Scientific Journals	Review, meta-analysis
*Methods*	Direct assessment of IMS: • experimental in-person setting • with human partner • using kinematic measures	Indirect assessment of IMS: • with non-human animations, digital characters, virtual partners • subjective measure of IMS (i.e., questionnaires, observational measures)
*Sample*	Presence of both control group and ASD group	Only ASD participants, neurotypical individuals with autistic traits or siblings

Far from being ascribable to mere imitation, IMS is defined by temporal and spatial contingency parameters that are not measurable by one or more observers’ offline coding. From this consideration derives the choice of including kinematic but not subjective measures (i.e., video coding made by an observer) but rather include precise measures of movement synchronization. Indeed, although a human observer could detect synchrony happening during interaction, the quantification of such subtle movement dynamics that characterize the construct of interest would be more accurate and precise if coded by a computer rather than a human. On the other hand, video-based and/or sensor-based tracking of kinematic patterns offer a more objective and precise method for assessing motor synchrony. Using technology and algorithms to track and analyze movement synchrony also reduces potential biases and subjectivity associated with human observer coding.

Moreover, studies were eligible for inclusion if the sample consisted of both an ASD and control group. The definition of control group as an inclusion criterion was intended to have at least two types of dyads to compare, which very often turned out to be TD-ASD vs TD-TD, but could have embraced ASD-ASD dyads as well. In fact, the idea is that in order to explore how synchrony works in autism we needed to compare it with non-autism. Notably, the presence of both ASD and TD groups in each study that was included is necessary for effect size calculation purposes. Unfortunately, only *n* = 1 paper compared TD-TD with ASD-ASD dyads (60) and we therefore excluded these data from our analyses to conduct the meta-analysis on a more homogeneous corpus of studies, whilst its content was included in our systematic review. The interaction dynamics are in fact hypothesized to be different here and to test this we would need more studies with this type of dyad and run moderation analyses, but unfortunately this was not the case. To minimize heterogeneity of populations, we excluded studies that recruited participants without ASD (i.e., siblings of ASD people or neurotypical individuals with autistic traits). As we were not interested in intervention effects, we included works that aimed at training IMS but used pre-treatment measures in our meta-analysis.

Crucially, it is necessary to find a balance between narrowing the focus on a specific construct and including studies that looked at that construct with different methods, or that focused on specific sub-components (e.g. spontaneous or instructed synchrony, with different partners). Motor synchrony occurs in a variety of contexts, which can vary from less to more naturalistic. As we will see, this is reflected in the variety of tasks adopted to study the phenomena. Across tasks and contexts, an important differentiation is made between “instructed” and “spontaneous” synchrony, which has been discussed in the literature and defined in the introductory part of our paper. We chose not to set constraints as for the type of task and type of synchrony since IMS itself encompasses a range of nuances, and all of them are of equal value in contributing to the big picture. If we did otherwise, we would miss useful information that would enable a better understanding of how the phenomenon of interest works. Given our interest towards the analysis of motor synchrony in human social interactions, we excluded studies assessing synchrony towards non-human animations, digital characters, or virtual partners, whilst including in-person human interactions.

**Figure 1:**
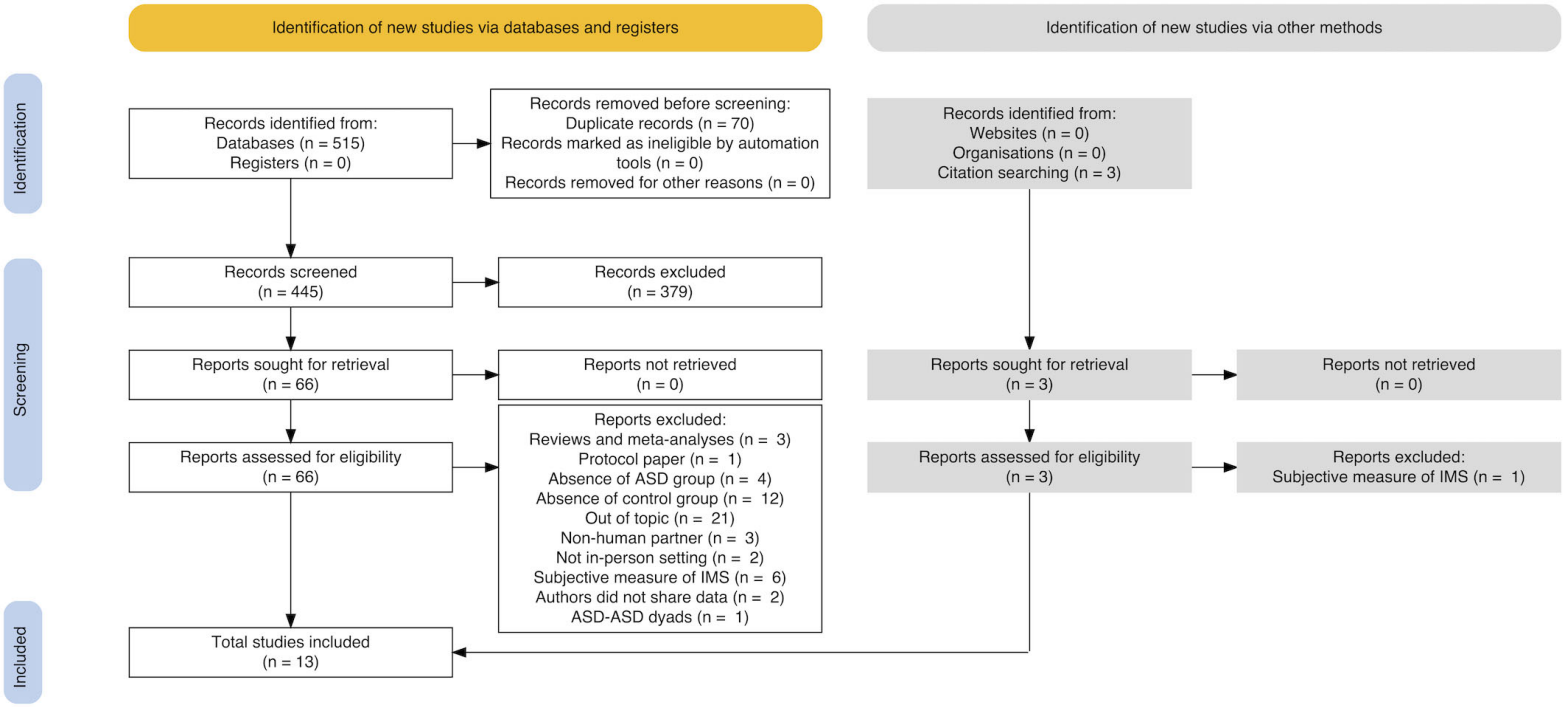
Flow diagram (following PRISMA guidelines).

The literature selection process is illustrated in the PRISMA flow diagram (**Figure 1**). Sixteen studies are included in the systematic review, while thirteen studies were ultimately selected for meta-analyses.

### 2.3 Data extraction

For each paper included, the following information was extracted and registered in a purposely built form:

- authors, publication year, DOI, country;
- sample size, M:F ratio, age (range, mean, standard deviation);
- means and standard deviations of outcome measure (IMS), separately for ASD and TD groups;
- experimental conditions, task(s) used to evaluate IMS, type of synchrony (spontaneous or instructed), number and type of measures.

All extracted information was recorded by the third author and checked by the second author. Some of the included papers did not contain all the information that was required to perform the analyses, thus corresponding authors were separately contacted by the first author to retrieve specific missing values.

Crucially, in many studies the authors presented the participants with batteries of tasks, not all of which aimed to assess interpersonal motor synchrony. For this reason, we considered data only from the tasks that were relevant for our research question.

Importantly, when the selected studies included measures of asynchrony and reaction times, a subsequent transformation of the values was necessary to ensure the meaningful interpretability of effect sizes [e.g., (61–63)]. Specifically, these measures were transformed to the negative of their absolute values, establishing a framework where lower numerical values corresponded to reduced synchrony. For instance, Yoo and Kim (62) measured asynchrony centered on the difference between the onset timing of tapping and cueing; consequently, values approaching zero denoted heightened synchrony, while both positive and negative values indicated varying degrees of asynchrony. To obtain a measure that was representative of synchrony and comparable to the others, we computed absolute values and subsequently reversed them to negative. Similarly, Fulceri et al. (61) specified that their measure of reaction times revolved around the difference, in milliseconds, between the Child Start Time and the Experimenter Start Time; hence, lower values in this context indicated heightened synchrony. Therefore, when handling reaction times (61, 63) or their coefficient of variation (61), we also applied negation of the absolute value, as higher values in this context would otherwise denote asynchrony rather than of synchrony.

### 2.4 Statistical approach and analyses

#### 2.4.1 Effect size

Multiple measures were adopted to assess IMS in each study, meaning a multitude of instruments and measurement scales being used to assess our outcome measure. As stated in [Borenstein et al. (64), pp.25], raw mean differences in this case would not be appropriate to combine the different results, while the Standardized Mean Difference (δ) would provide an effect size index that is comparable across studies. Given the low to medium sample size of the considered studies, to estimate δ we used the Hedges’ *g*, an unbiased estimator that gives less distort results than the usual Cohen’s *d* [(64), pp.27]. To calculate the Hedge’s g, the ‘*metafor::escalc*’ function was used (65). To this point, each study had multiple effect sizes. These reflect a variety of collected measurements that relate to multiple angles of observation of the same phenomenon of interest capturing several different facets. However, considering these effect sizes separately would not be appropriate, as the measurements they come from are interdependent (i.e., same study). For this reason, it becomes necessary to aggregate the effect sizes into a composite one that quantifies our outcome measure weighting for the multiplicity of measures that contribute to it. To account for the non-independency of the effect sizes within each study, we computed an aggregated effect size for each study adopting the method suggested by [Borenstein et al. (64), Chapter 24]. To this end, we used the *‘Mad::agg’* function (66). To use this method the correlation among outcomes should be known. As this was not the case for our selected studies, we ran three meta-analyses hypothesizing three plausible correlations to evaluate whether our results changed accordingly, as suggested by Borenstein et al. (64). The same method was used, for example, in Benavides-Varela et al. (67). Given that the outcomes that were aggregated within each study were similar from a theoretical point of view it seems conceivable to us that they correlate strongly. We indicated as most plausible the value of *r* = .50 based on Cohen (68) guidelines, in which a correlation effect of *r* = .50 is referred to as a large effect and therefore we hereby report analyses on this specific case. The same set of analyses with *r* = .30 and *r* = .70 can be found in the **Supplementary Materials**.

#### 2.4.2 Statistical analyses

The analyses were conducted with R software (69). In particular, the *‘metafor’* package (65) was used to perform the meta-analysis and the subsequent analyses to deepen the obtained results. A random-effects meta-analytical approach was chosen due to the number of different laboratories involved and the high variability in effect size indices. After running the random-effects models, we explored the heterogeneity between studies through inspection of forest plot and evaluation of the Q-statistics (70). Heterogeneity between the studies is indicated by significant values of Q-statistics, which are distributed like the chi-square under the null hypothesis of homogeneity. To evaluate the extent of heterogeneity, we considered the *I^2^* (71) and τ indexes, as well as the prediction intervals (72). Although *I* ^2^ is commonly reported in meta-analyses as a measure of heterogeneity, it does not tell us how much the true effects vary but what proportion of the variance in the observed effects reflects variance in true effects rather than sampling error (73). The estimated amount of total heterogeneity underlying distribution of true effect sizes is provided by τ^2^, the between-study variance of the effect size across the population of studies. Importantly, by looking at τ (squared root of estimated τ^2^) we can quantify the between-study standard deviation. Complementarily, the prediction interval indicates the range of value within which the true effect of a new and unique study will fall in 95% of cases (74).

#### 2.4.3 Sensitivity analysis and evaluation of publication bias

Various model diagnostic procedures are available to identify outliers and/or influential studies, and for conducting sensitivity analyses, which are recommended to assess whether the findings are robust to the decisions made in the process of obtaining them (75). As the main objective of a meta-analysis is to provide a reasonable summary of the effect sizes of a body of empirical studies, the presence of such outliers may distort the conclusions of a meta-analysis. Moreover, if the conclusions of a meta-analysis hinge on the data of only one or two influential studies, then the robustness of the conclusions are called into question (76). The evaluation of outliers and influential cases is particularly useful in cases like ours that are characterized by a low number of studies and where, therefore, even a single study can have a strong effect on the estimated effect-size. Since a single outlying study could be the source of heterogeneity, a recommended method to identify suspicious cases is the leave-one-out method, which consists in repeatedly fitting the specified model, leaving out one study at a time. Using the*’leave1out’* function we explored to which extent model parameters change when each study is removed from the sample. The basic rationale behind identifying influential data is that when single units are omitted from the data, models based on these data should not produce substantially different estimates. Furthermore, we used Cook’s distance to estimate the influence of each data point on model parameters, evaluating influential cases. More specifically, Cook’s distance provides further information to identify influential data points and complements the information collected by the previously mentioned method as it quantifies the change in the meta-analysis parameters when each single study is removed from the sample (indeed, high values of Cook’s distance indicate strong influence on meta-analysis parameters). The sensitivity analysis we conducted provides a more informative picture for the reader by transparently clarifying the weight each study has on the final effect size estimate. We further evaluated the publication bias by means of the funnel plot and using the trim and fill method, a nonparametric data augmentation technique which estimates and adjusts the estimates for the potential number and effect size of missing studies (77, 78).

## 3 Results

### 3.1 Systematic review

Although studies show that humans have a natural propensity to align their behavior during interactions (*for review see* 2, 6, 13), the processes by which IMS is achieved could be more or less explicit. The distinction between spontaneous and instructed synchrony has been recently scrutinized by Howard et al. (79), whose findings underscored that while instructions failed to enhance synchronization accuracy in adults, they did improve accuracy among children. One could wonder whether this also applies to the body of literature in the autism field. On the one hand, instructions should support synchrony as driving participants’ attention to the process itself and thus encouraging an active effort to achieve it, but they could also be an obstacle whereby verbal comprehension is limited. On the other hand, spontaneous paradigms would shed light on low-level synchronization abilities observable across the whole spectrum.

In the following subsections we delve into the *n* = 16 studies that were eligible for review. First, we separately delineate the empirical findings pertaining to spontaneous and instructed IMS in dyads where at least one member is in the autism spectrum; second, we summarize the measures that were employed to assess synchrony across the studies. A schematic overview of each study can be found in **Table 2**.

**Table 2:** Overview of the studies included in the systematic review.

Authors, Year	Task	Measure	Dyads	Type of Synchrony	Take home message
Brezis et al. (2017) (80)	Mirroring each other while moving handles along parallel tracks: alternate leading, following, or joint improvisation with no pre-specified roles	Percentage and duration of Co-Confident periods	Adult-adult	Instructed	ASD participants showed less periods of synchrony, particularly in the follower role, compared to when they were leading, or no specific roles were established. Overall, they also showed shorter periods of synchrony. General motor abilities among ASD participants accounted for some, but not all, of their reduced synchrony in the follower role. General social skills did not predict IMS levels.
Chen et al. (81)	Preschoolers were invited to play with their teacher using some toys such as blocks, a set of toys for cooking, and two magnetic robots	Windowed cross-correlation of body movements time series (head, trunk, right arm) computed by means of an automated human pose estimator. Based on videos, the authors segmented episodes of two-ways interaction from one-way adult engagement only.	Child-teacher	Spontaneous	Diminished synchrony was observed in both TD and ASD when only adults exhibited social engagement compared to situations where both adults and children interacted. In two-way interactions, the ASD group displayed decreased IMS in the upper body and trunk compared to the TD group, whereas during one-way adult engagement, the ASD group exhibited heightened IMS in the head.
Delaherche et al. (82)	Participants had to build a puppet from multiple elements together with their therapist, in three conditions: when seeing the therapist performing actions, when hearing instructions on how to put the puppet together, when giving instructions.	Windowed cross-correlation of motion energy time series (ROIs: child, therapist - each had global, posture, hands regions)	Child-adult	Instructed	The presence of a folding screen obstructing others’ sight in the conditions where the child only heard or gave instructions made synchronization harder especially for the ASD children, in fact the TD group tended to be more in sync with the therapist’s movement despite the folding screen.
Fitzpatrick et al. (2013) (83)	Social synchronization: the experimenter demonstrates several movements directed to objects, own body or space, then asks the child to do them together	Relative phase to calculate the frequency of occurrence in each relative phase region	Child-adult	Instructed	The ASD group showed reduced simultaneous synchronization only in object-directed movements. There is no clear evidence on whether IMS is impaired in autism.
Fitzpatrick et al. (2016) (84)	Pendulum coordination paradigm: adolescents swung the pendulum with the dominant hand while facing the parents swinging the pendulum with the non-dominant hand	Circular variance of relative phase	Adolescent-parent	Instructed	ASD adolescents showed less synchronization in both spontaneous and intentional interpersonal coordination.
Fitzpatrick et al. (2017) (85)	Social synchronization: the experimenter demonstrates several movements directed to objects, own body or space, then asks the child to do them together	Weighted coherence from the time series movements of the child and experimenter	Child-adult	Instructed	ASD children exhibited lower social synchronization ability than TD children in all types of social motor synchronization tasks. The ASD group performed drumming movements that were slower and more variable in both spacing and timing than TD.
Fulceri et al. (2018) (61)	Cooperative joint action: the child had to move their arm to insert a “banana” coin into a “monkey” box that was moved by the experimenter, thus coordinating with the experimenter’s movement.	Reaction times, Coefficient of variation of reaction times, Movement time, Asynchrony of reaching	Child-adult	Spontaneous	ASD children showed reduced coordination with the adult as captured by some kinematic parameters, especially when the final destination of the movement was not known beforehand.
Georgescu et al. (2020) (86)	Guided conversations (i.e., a cooperative or competitive conversation on an instructed topic)	Windowed cross-lagged correlations of the motion energy time series (ROIs: head and body of each participant)	Adult-adult	Spontaneous	In a conversational setting, dyads with at least one ASD participant, compared to TD-only dyads, showed reduced interpersonal motor synchrony. This was not due to the quantity of movement produced, which did not differ between groups.
Glass et al. (2023) (60)	Tablet-based games where participants had to cooperate to find the matching colors in a series of dots (Colors, designed with no additional design features to facilitate collaboration) and match and sort pictures based on their categories (Connect, designed to support collaboration by facilitating engagement and other-awareness)	Windowed cross-lagged correlations of the motion energy time series	Child-child	Spontaneous	In both the shared tablet activities — Connect and Colors — the neurotypical group exhibited comparable motor synchrony to the autistic group in Colors, yet demonstrated lower IMS in Connect. Interestingly, the autistic group maintained similar IMS levels across both activities, indicating that within specific social contexts and task types, autistic children exhibit comparable or even heightened synchronization abilities compared to neurotypical children.
Kawasaki et al. (2017) (87)	Social synchronization: participants were instructed to tap two keys back and forth at a time interval equal to that of the partner. The tapping tempo was not predetermined nor directed	Rates of synchronized tapping (based on tapping intervals)	Adult-adult	Instructed	ASD adults had lower rates of synchronization with TD partners. Synchronization rate was correlated with autism severity. Differences in theta activity measured by EEG were also found in the ASD group. Potential associations between theta activity, synchronization rate, and symptom severity are discussed.
Kruppa et al. (2021) (63)	Computer based game, in which participants had to cooperate or compete to press a button and make a dolphin jump, and catch the ball	Mean of the absolute differences in response times of each dyad	Child-parent, child-stranger	Instructed	Overall, higher synchrony occurred during competition compared to cooperation, and with the stranger compared to the parent. ASD children were less synchronous than TD children, across conditions and partners. No group differences were observed at the neural level (wavelet coherence from fNIRS signals).
Lampi et al. (2020) (88)	Interpersonal hand-clapping	Weighted coherence from the time-series movements	Child-adult	Spontaneous	In the ASD group, poorer IMS was associated with higher levels of Restricted and Repetitive Behaviors.
Liu et al. (2021) (56)	Caregiver-child dyads are involved in a musical (song) and non-musical (picture) book-sharing activity	Windowed cross-correlation of motion energy time-series (ROIs: child, adult)	Child-caregiver (either parent or other)	Spontaneous	ASD children showed lower motor synchrony with their caregiver compared to their typically developing peers, regardless of the shared book being musical or non-musical.
Marsh et al. (2013) (89)	A parent read a storybook to the child while sitting in their own rocking chair and rocking throughout to a set tempo. Children sit on their own rocking chairs while listening.	Continuous relative phase to calculate the average amount of time the dyad spent in a given relative phase	Child-parent	Spontaneous	ASD children exhibited significantly less in-phase rocking with their parents than TD children, thus showing reduced spontaneous synchronization. The authors argue that unintentional low-level motor synchronization could contribute to core impairments observed in autism (i.e., engage in joint attention, joint action, and mimicry)
Noel et al. (2018) (90)	Non-verbal synchrony (i.e., head, hand, trunk) during seated neuropsychological testing and natural conversation	Pearson correlation of motion energy time-series (ROIs: head, hand, trunk of each participant)	Child-adult	Spontaneous	ASD children, compared to TD, showed reduced motor synchrony with the adult, and reduced complexity for head and hand movements. However, no correlations were found between interpersonal synchrony and the complexity of movements, multisensory integration, or general movement characteristics.
Yoo et al. (2018) (62)	Participants were instructed to drum on a pad while the experimenter was also drumming on a separate pad. A rhythmic auditory cue was available to the participant in one condition.	Asynchrony (synchronization errors) measured by calculating the difference between the onset timing of tapping and the onset timing of the cueing in milliseconds	Child-adult	Spontaneous	When comparing a baseline ability to synchronize to an auditory rhythmic cue, ASD and TD perform similarly. An overall reduced ability to synchronize in the interpersonal condition is found in ASD compared to TD, however in both groups synchronization improved when the interpersonal condition was also accompanied by a rhythmic auditory cue. The highest variability was observed in ASD during the interpersonal synchronization task without auditory cueing.

#### 3.1.1 Spontaneous IMS

In a dyadic and naturalistic situation with a neurotypical adult, preschoolers and children with ASD exhibited reduced synchronization compared to their neurotypical peers (56, 61, 62, 81, 88, 89). Activities could include story reading, drumming, hand clapping or cooperative games, which were purposely set for children to be engaged in an ecological interactive setting. Reduced spontaneous synchronization was also observed during seated neuropsychological testing and natural conversation in children (90) as well as in adult conversations (86). The ability to synchronize with the interaction partner also seems to be negatively correlated with autistic traits from early ages. In fact, Chen et al. (81) found that autistic traits especially in the areas of social skills, communication, and attention switching measured in the ASD sample are associated with reduced IMS.

One could argue that the quantity and quality of movements might explain the differences found between groups. Relatedly, Noel et al. (90) measured movement complexity, duration, and quantity during the task to shed light on specific patterns of motion. Although higher complexity (i.e., non-stereotyped, non-rhythmic, and not easily predictable) of movements was found in the TD vs ASD group, no correlation emerged between IMS and any of the examined factors (90). In line, Georgescu et al. (86) found that the reduced alignment if the dyad included at least one ASD participant was not due to the quantity of movement produced, which did not differ between groups (86). This corpus of research suggests intra-individual atypicalities in motor abilities not to be enough to explain the reduction in IMS observed in ASD-TD dyads. Differences in movement planning could on the other hand influence IMS. In fact, in a cooperative joint action task children with ASD showed reduced coordination with the adult especially when the destination of the movement was not known beforehand, suggesting that they struggled more to achieve IMS when having to rely solely on kinematic features of other’s movement in the absence of a visual goal (61).

Diminished attention toward the interaction partner could weaken IMS. Intuitively, the visual anchor of the other supports IMS as allowing continuous monitoring and adjustment of one’s movements to the counterpart’s. In fact, studies show enhanced IMS when visibility of the partner is optimal and decreased when it is not (81, 84). Crucially though, the content of the activity could scaffold social attention itself. For instance, when engaged in a book sharing activity with their caregivers, ASD children devoted less visual attention to the other their TD peers, but this was attenuated when using a musical book rather than a picture book, suggesting musical activity to scaffold mutual engagement (56). Similarly, Glass and Yuill (60) found that the level of IMS varied across groups based on the task type. They involved ASD-ASD and TD-TD children pairs in two joint tablet-based activities and found that the two groups exhibited comparable IMS in the one activity that was not designed to deliberately stimulate interaction, while demonstrating lower IMS in the one activity that was designed to encourage collaboration and other-awareness. Notably, autistic children maintained consistent synchronization levels across both activities, suggesting that in specific social contexts, they may possess comparable or heightened synchronization skills (60). To our knowledge, only one other study looked at ASD-ASD dyads compared to ASD-TD and TD-TD, but did not find an advantage of neurodevelopmentally homogeneous (over heterogeneous) dyads on IMS (86). Given the controversial results of these two pivotal studies (60, 86) further research is needed to examine whether IMS in ASD-ASD dyads is or is not enhanced, and which factors contribute to such differences.

#### 3.1.2 Instructed IMS

Motor synchrony is not only a spontaneous mechanism that facilitates interactions with each other on an implicit level, but it is also a state that can be pursued intentionally and consciously, enabling us to achieve specific goals in shared activities such as sports, singing and music, as well as play and everyday activities. Literature suggests that this ability to voluntarily synchronize with others is reduced in people with ASD from childhood to adulthood (63, 80, 82–85, 87).

Some studies asked children to perform a series of actions (either directed to objects, their body, or space) together with the experimenter and suggested that the target of a movement might also play a role in one’s ability to synchronize. In fact, children with ASD showed lower synchronization abilities only in object-directed movements (83). However, a further implementation of the same experiment found reduced synchronization in all types of IMS tasks for the ASD compared to TD children (85). The ability to voluntarily synchronize with others has also been found decreased in adolescents with ASD (84) and seems to persist into adulthood. When instructed to tap two keys back and forth at the same tempo as that of the partner’s, adult dyads with one ASD participant showed lower rates of synchronization (87). In line with literature on spontaneous IMS, movement features do not seem enough to explain reduced voluntary synchrony. Although slower and more variable movements in both spacing and timing were observed in ASD, this only partially related to IMS (85). Similarly, Brezis et al. (80) found that general motor abilities among participants with autism accounted for some, but not all, of their reduced synchrony especially when situated in the follower role. In the same study, results highlight shorter periods of synchrony in ASD compared to TD dyads, regardless of leading, following, or having no pre-specified role within the interaction (80).

Ultimately, a first effort has been done to explore the neural dynamics that underpin IMS during instructed interaction, although results appear controversial. More specifically, Kruppa et al. (63) used fNIRS to explore brain-to-brain dynamics during a joint task (cooperative and competitive) and found no group differences although lower IMS emerged behaviorally for the ASD group. When looking at individual neural activity with EEG during a cooperative tapping task, Kawasaki et al. (87) found higher theta-activity in the frontal cortex in ASD, that was however related to severity of ASD and difficulty to adapt to other’s irregular behaviors rather than performance on the task itself. Further research is necessary to shed light on the neural underpinnings of IMS, especially in autism.

#### 3.1.3 Description of measures adopted to assess IMS

##### 3.1.3.1 Continuous relative phase

This measure is used in Fitzpatrick et al. (83, 84) and Marsh et al. (89). It describes the phase space relation between two segments as it evolves throughout the movement [for review, (91)]. More specifically, it measures the angle between two rhythms in a cycle, meaning that identical movements have 0° relative phase (in-phase) and opposite movements have 180° phase (anti-phase). This is done throughout the duration of a movement, resulting in a time series. In Fitzpatrick et al. (83) 9 relative phases were defined (from 0° to 180°, by 20°) and their frequency of occurrence (in percentage) has been computed to evaluate the predominance of in-phase or anti-phase movements. A similar approach has been used in Marsh et al. (89): each rocking segment was weighted by its relative length and 9 relative phases were considered, in 20° increments arrayed from in-phase (10° either side of 0°) to anti-phase (10° either side of 180°). From this measure, the authors calculated the average amount of time a dyad spends in a given relative phase. Continuous relative phase was also used in Fitzpatrick et al. (84) to further compute the circular variance, which indicated the proportion of same relative phases in the two individuals’ time series. The circular variance of continuous relative phase is a proportion on a 0-1 continuum where 1 means perfect synchrony and 0 absence of synchrony.

##### 3.1.3.2 Motion energy

Motion energy is an automatic frame-differencing method to quantify movement dynamics by detecting the amount of pixel changes between consecutive frames (92). One or more regions of interest (ROIs) in which motion energy is computed are usually identified. This measure is used in Liu et al. (56), Georgescu et al. (86), Noel et al. (90) and Glass and Yuill (60). Different ROIs have been selected by different groups: Liu et al. (56), as well as Glass and Yuill (60) selected 2 ROIs only (corresponding to each person) as being interested in a whole-body coordination, while other groups chose to differentiate between multiple ROIs within each individual [i.e., head and body in Georgescu et al. (86); head, hand, trunk in Noel et al. (90)]. After motion energy is extracted, researchers aiming to assess the strength of the relationship between the two individuals’ movements commonly calculate a correlation. Among the studies included in the present meta-analysis, the correlation can either be a Pearson correlation for each epoch (~30s) or a windowed cross-correlation of time series (~5s window) usually followed by a Fisher’s transformation to allow between-conditions comparisons. Correlation coefficients are then averaged to obtain a final value rather than one value for each time segment.

##### 3.1.3.3 Weighted coherence

The weighted coherence has been defined as the proportion of shared variance between two systems over an entire band of frequencies (93) and thus consists in the estimated correlation between partners’ movements. This measure is used in Lampi et al. (88) and Fitzpatrick et al. (85). In both studies, motion tracking systems were used, and a correlation coefficient has been computed throughout the time series across the frequency band from 0 to 2 Hz. As the authors stated, the weighted coherence is a weighted average measure of the correlation of the two time-series across this frequency range and spans between 0 and 1 (85). The more values are close to zero, the less dyad is in-synch; conversely, values close to 1 increasingly indicate synchrony of movements.

##### 3.1.3.4 Other measures

Brezis et al. (80) analyzed the percentage and duration of co-confident periods, that are motion segments lasting from 0.2 to 8 sec during which high synchrony with little jitter (i.e., smooth movement) is observed. Fulceri et al. (61) adopted three different measures to assess the extent to which dyads manifested movement synchrony: reaction times, coefficient of variation of reaction times and asynchrony of reaching. The first refers to the difference between child and experimenter’s movement start time; the second corresponds to the ratio between the standard deviation and the mean of the reaction time; the third is the difference between the child and experimenter’s movement duration; the last consists in the time delay between experimenter and child’s movement end time. Similarly, Yoo and Kim (62) used the difference between participant’s onset of tapping and external cueing (i.e., other’s onset of tapping in their interpersonal synchrony condition), while Kruppa et al. (63) averaged the absolute differences in response times of each dyad. Another measure of synchrony that has been used by Kawasaki et al. (87) consists of rates of synchronized tapping.

### 3.2 Meta-analysis

#### 3.2.1 Participant details and descriptive statistics of included studies

Descriptives of the selected works can be found in **Table 3**. All the included studies were conducted between 2013 and 2021. The age range considered spans from children to adolescents and adults. Most research came from the USA (*n* = 8 out of 13) and involved a participant-experimenter interaction (*n* = 7), while some studies involved caregiver-child (*n* = 4) or a participant-participant (*n* = 2) dyad. Only one study further included a child-stranger dyad as a separate condition from parent-child. The type of synchrony investigated also varies (*n* = 7 spontaneous, *n* = 6 instructed).

**Table 3:** Descriptive statistics of the studies included in the meta-analysis.

ID	Authors	Country	Group	*n* dyads	Interaction partner	M/F ratio	Age				Type of Synchrony	es	var
							range	mean	sd	values refer to			
1	Brezis et al. (80)	Israel	Control	35	experimenter	28:7	19 - 45	25.9	6.37	*n* = 69 adults (*n* = 35 TD and *n* = 34 ASD)	instructed	0.66	0.04
			ASD	34		31:3	20 - 45	28.6	6.26				
2	Fitzpatrick et al. (83)	USA	Control	3	experimenter	1:2	4 - 5.6	4.8	0.75	*n* = 8 children (*n* = 3 TD, *n* = 5 ASD)	instructed	0.21	0.34
			ASD	5		4:1	5 - 7.4	6.21	1.17				
3	Fitzpatrick et al. (85)	USA	Control	27	experimenter	21:6	6.33 - 10.8	8.24	1.46	*n* = 50 children (*n* = 27 TD, *n* = 23 ASD)	instructed	1.00	0.05
			ASD	23		20:3	6.08 - 10.75	8.08	1.44				
4	Fitzpatrick et al. (84)	USA	Control	9	parent (*n* = 18)	7:2	12 - 16	14.44	1.13	*n* = 18 adolescents (*n* = 9 TD, *n* = 9 ASD)	instructed	0.34	0.13
			ASD	9		8:1	12 - 17	13.67	1.94				
5	Fulceri et al. (61)	Italy	Control	11	experimenter	9:2	6.3 - 9.8	7.57	0.71	*n* = 22 children (*n* = 11 TD, *n* = 11 ASD)	spontaneous	0.88	0.11
			ASD	11		10:1	5.11 - 10.3	7.82	1.32				
6	Georgescu et al. (86)	Germany	Control	10	other participants	17:12	33 - 51	41.8	8.86	average dyad values of TD-TD (*n* = 20 TD) and ASD-TD (*n* = 9 ASD, *n* = 9 TD)	spontaneous	0.83	0.12
			ASD	9		5:4	30 - 51	40.72	10.45				
7	Kawasaki et al. (87)	USA	Control	24	other participants (TD had two sessions)	12:12	18.9 - 32.1	25.6	6.6	*n* = 48 adults (*n* = 24 ASD and *n* = 24 TD)	instructed	0.83	0.09
			ASD	24		14:10	22 - 36.4	29.2	7.2				
8	Kruppa et al. (63)	Germany	Control	41	parent (*n* = 59), stranger (*n* = 32)	18:23	8 - 18	12.66	2.79	*n* = 59 children (*n* = 41 TD, *n* = 18 ASD)	instructed	0.62	0.06
			ASD	18		18:0	8 - 18	13.54	2.96				
9	Lampi et al. (88)	USA	Control	47	experimenter	34:13	6 - 10	7.85	1.49	*n* = 97 children (*n* = 47 TD, *n* = 50 ASD)	spontaneous	0.83	0.03
			ASD	50		34:7	6 - 10	8.02	1.44				
10	Liu et al. (56)	USA	Control	16	caregiver (*n* = 29)	10:6	1.66 - 4.33	2.99	0.7	*n* = 29 preschoolers (*n* = 16 TD, *n* = 13 ASD)	spontaneous	3.78	0.11
			ASD	13		10:3	1.75 - 5.75	3.88	0.85				
11	Marsh et al. (89)	USA	Control	7	parent (*n* = 14)	4:3	2.8 - 4.6	3.75	0.12	*n* = 14 children (*n* = 7 TD, *n* = 7 ASD)	spontaneous	0.21	0.31
			ASD	7		5:2	3.8 - 4.1	3.94	0.74				
12	Noel et al. (90)	USA	Control	15	experimenter	11:4	8.9 - 14.5	10.94	2.13	*n* = 27 children (*n* = 15 TD, *n* = 12 ASD)	spontaneous	0.24	0.11
			ASD	12		8:4	7.9 - 16.5	12.2	3.75				
13	Yoo et al. (62)	Korea	Control	42	experimenter	23:19	11 - 16	13.5	0.8	*n* = 52 children (*n* = 42 TD, *n* = 10 ASD)	spontaneous	0.44	0.08
			ASD	10		10:0	11 - 16	13.4	1.4				

Note that the number of dyads refers to the statistical units. For instance, Kruppa et al. (63) had children participants interacting with one of their parents and a stranger. Only children’s age contributed to the values reported in the table and the two interactions were treated as separate conditions in the present analysis. In two cases, adult participants interacted with each other and therefore the age values reported here refer to all the people involved in interactions. The age values reported for Georgescu et al. (86) are dyadic, meaning that they come from the average age values of the two components for each pair. Those reported for Kawasaki et al. (87), refer to the TD and ASD participants separately as detailed in the table.

#### 3.2.2 Random-effects model meta-analysis

**Figure 2:**
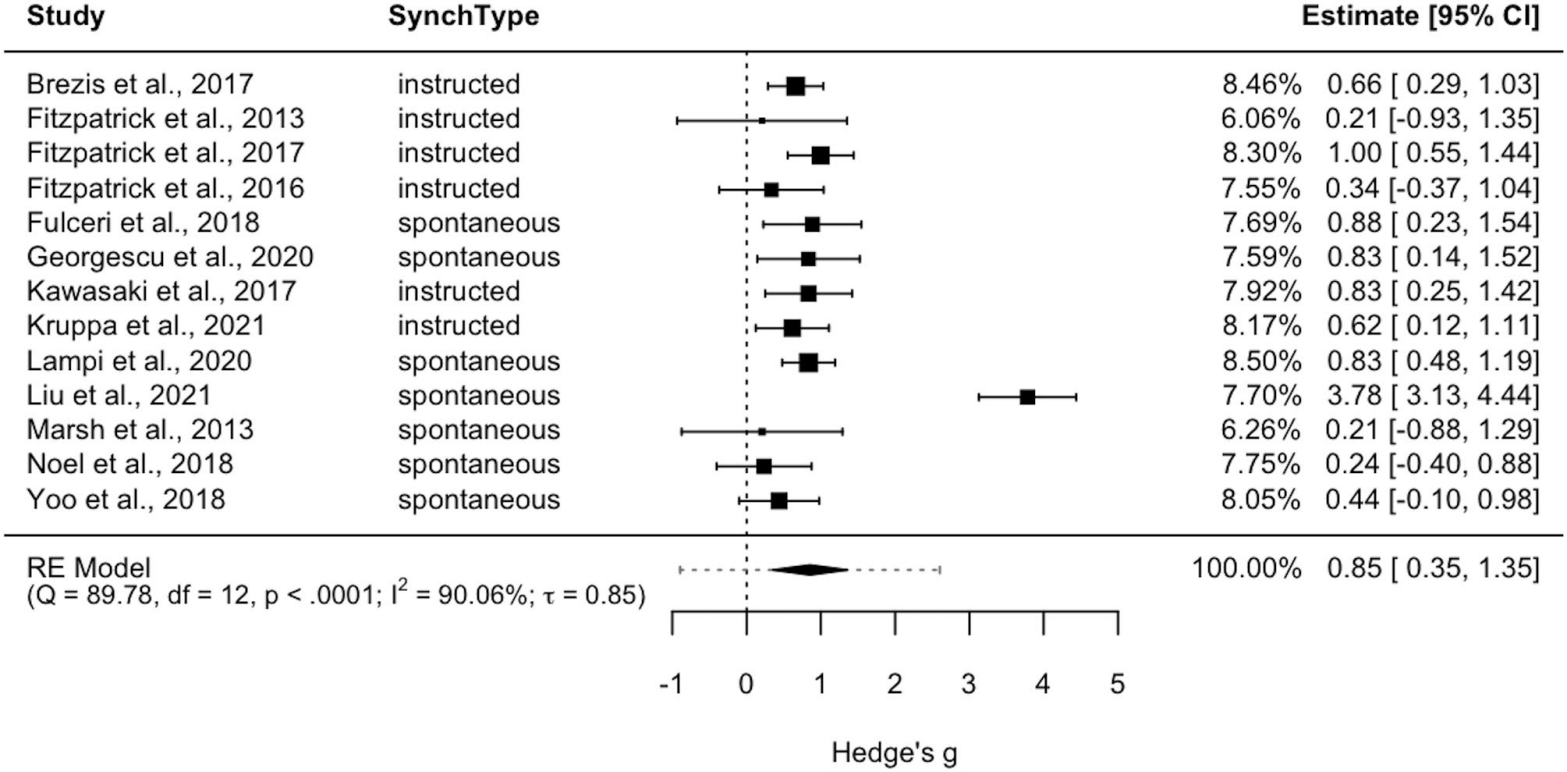
Forest plot for random-effects model meta-analysis. Note that positive Hedges’ g indicates higher IMS in the comparison group (TD-TD dyads). The dotted line represents the prediction interval.

The random effects meta-analysis showed a large aggregated effect size (*Hedge’s g* = .85, *p* < 0.001, 95% *CI*[.35, 1.35], 95% *PI*[-.89, 2.60]). Although the estimated effect size could be considered large, the wide width of the confidence interval suggests that also small to medium effect sizes should be considered consistent with the observed data. The heterogeneity was significant *Q*(12) = 89.78, *p* <.0001, with high values of the *I^2^* index (*I^2 ^= *90.06%), τ = 0.85 and *PI*[-.89,2.60]. Importantly, whilst *CI* quantifies the accuracy of the mean and indicates where the mean effect is likely to be, *PI* quantifies the dispersion (or distribution) of effect estimates (73, 74). In our case, if 95% *CI*[.35, 1.35] indicates the uncertainty around the estimate of the average effect of IMS across the two populations, 95% *PI*[-.89,2.60] indicates this as the range of value within which the true effect of a new and unique study will fall in 95% of cases, further highlighting the variability of the phenomena of interest. This is also evident from the is between-study standard deviation we found (τ), which is particularly high when compared to the values commonly reported in meta-analysis pertaining to the psychology domain (94). In **Figure 2**, forest plot shows the aggregated effect sizes for each study as well as the estimate of the common effect size and the prediction interval.

A moderation analysis on the random-effect model was performed to explore whether the type of synchrony (spontaneous vs instructed) influenced effect size estimates. No statistically significant results emerged: *QM*(1) = 0.64, *p* = 0.42.

The set of analyses hereby presented are in line with those conducted with the other hypothesized correlations of *r* =.30 and *r* =.70, which results are summarized in **Table 4** (see *SM for details*).

**Table 4:** Summary of results from the three meta-analyses (see SM for details).

Correlation	Hedge’s g	*I* ^2^	τ	*CI*	*PI*
*r* = .30	0.85	92.19%	0.86	[0.35;1.35]	[-0.91;2.61]
*r* = .50	0.85	90.06%	0.85	[0.35;1.35]	[-0.89;2.60]
*r* = .70	0.86	88.11%	0.85	[0.36;1.36]	[-0.88;2.59]

#### 3.2.3 Sensitivity analysis and evaluation of publication bias

**Figure 3:**
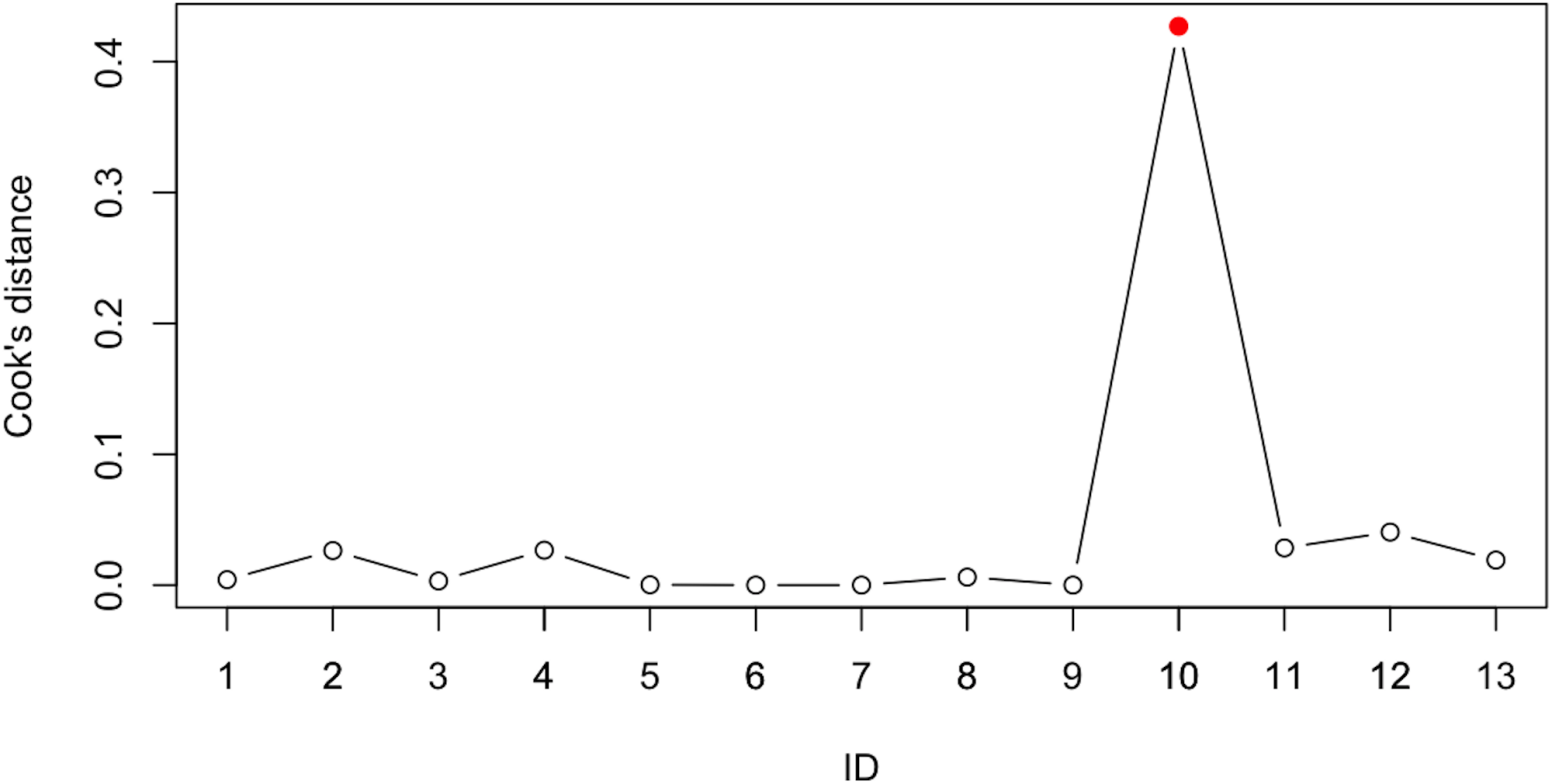
Sensitivity analyses with Cook’s distance. Note that IDs follow alphabetical order of included studies. See **Table 1** for specifications.

Sensitivity analysis using the leave-one-out method was performed to evaluate influential studies that may distort the effect size estimate. Results are reported in **Table 5** and depict the study from Liu et al. (56) as more influential than others, decreasing the effect size estimate. In line, Cook’s distance indicates that the study from Liu et al. (56) is away from the others (**Figure 3**). Although heterogeneity would importantly decrease if excluding this study, the effect size estimate would still be considered large and the confidence interval would nevertheless include high values, therefore we decided not to exclude the study.

**Table 5:** Sensitivity analyses with Leave-One-Out method.

Authors	Hedge’s g	*I* ^2^	τ	*CI*	*PI*
Brezis et al. (80)	0.87	90.08	0.90	[0.32; 1.42]	[-0.98; 2.72]
Fitzpatrick et al. (83)	0.89	91.03	0.88	[0.37; 1.42]	[-0.90; 2.69]
Fitzpatrick et al. (84)	0.90	90.89	0.88	[0.36; 1.43]	[-0.92; 2.71]
Fitzpatrick et al. (85)	0.84	90.54	0.90	[0.29; 1.39]	[-1.01; 2.69]
Fulceri et al. (61)	0.85	91.12	0.90	[0.31; 1.39]	[-1,00; 2.69]
Georgescu et al. (86)	0.85	91.15	0.90	[0.31; 1.40]	[-0.99; 2.70]
Kawasaki et al. (87)	0.85	91.00	0.90	[0.31; 1.40]	[-0.99; 2.70]
Kruppa et al. (63)	0.87	90.69	0.90	[0.33; 1.42]	[-0.97; 2.72]
Lampi et al. (88)	0.85	90.00	0.91	[0.31; 1.40]	[-1,00; 2.71]
Liu et al. (56)	0.69	0.00	0.00	[0.53; 0.84]	[0.53; 0.84]
Marsh et al. (89)	0.90	91.01	0.88	[0.37; 1.42]	[-0.90; 2.69]
Noel et al. (90)	0.90	90.67	0.88	[0.37; 1.44]	[-0.89; 2.70]
Yoo et al. (62)	0.89	90.68	0.89	[0.35; 1.43]	[-0.94; 2.72]

**Figure 4:**
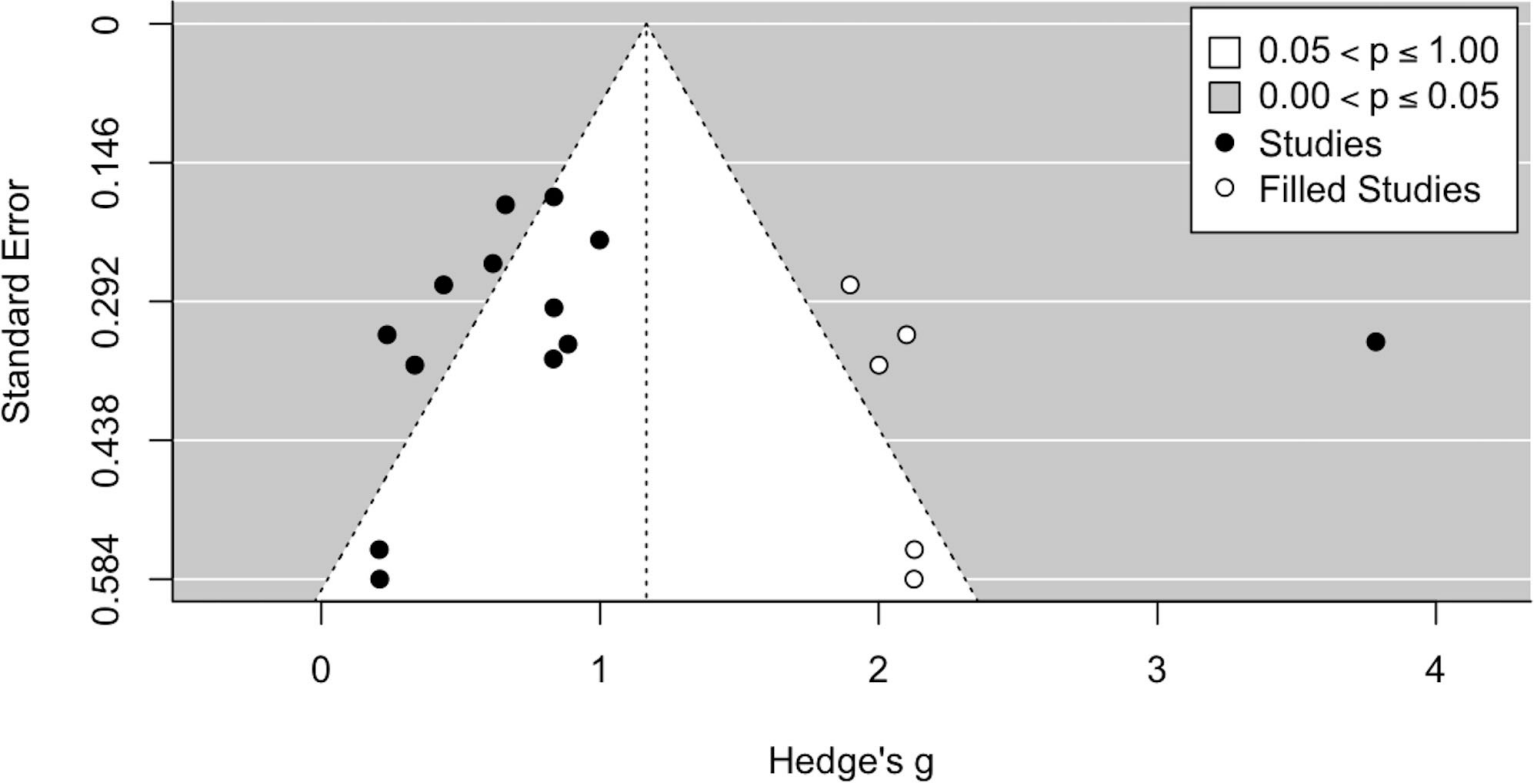
Funnel plot with trim-and-fill method.

To manage publication bias statistically, we graphically analyzed the funnel plot, drawn after the trim-and-fill method was implemented (**Figure 4**).

When accounting for publication bias via the trim-and-fill method, an even larger effect size (*Hedge’s g* = 1.17, *p* < 0.001, 95% *CI*[.73, 1.60]) was observed. Also, significant heterogeneity emerged Q(17)=130.30, p<.0001, with high values of the *I^2^* index (*I^2^* = 89.40%), τ = 0.88 and *PI*[-.60, 2.94]. The added studies trend in the direction of the hypothesized effect, contrary to the expectation in case of publication bias. Hence, in this case taking publication bias into account even increases the effect size estimate. This may be due to the relatively limited number of studies considered, which does not allow for an appropriate assessment of the publication bias, and especially to the study by Liu et al. (56), which has an extremely high effect size. Overall, results from the trim and fill methods do not rule out the presence of publication bias, although they should be taken with caution.

We further performed a sensitivity analysis to evaluate whether our results changed according to several imputed plausible correlations, as suggested by Borenstein et al. (64). The same method was used, for example, in Benavides-Varela et al. (67). Importantly, all three correlations and the corresponding three meta-analyses (respectively, *r* = .30, *r* = .50 and *r* = .70) ultimately lead to essentially the same results as shown in the **Supplementary Material**. In conclusion, although we find the correlation of.50 to be the most plausible, the results remain the same even when imputing the other correlations, thus indicating the robustness of our results.

## 4 Discussion

Our results suggest there are indeed differences in the extent to which ASD-TD compared to TD-TD dyads synchronize at a motor level during interpersonal interactions. Although this is consistent across studies and supported by the large effect size that emerged from the meta-analysis, the examined literature also depicts a multitude of different tasks and measures used to capture IMS in a quite broad age range and with different interactive partners. Interestingly, the confidence and prediction intervals from the meta-analysis suggest that small to medium effect sizes could also be plausible, highlighting the relevance of discussing the possible reasons for such uncertainty in spite of a clear direction of the effect. In the following subsections, we will thoroughly investigate potential sources of heterogeneity in our results, drawing upon findings included in our systematic review. This comprehensive exploration aims to provide insights into the factors contributing to variations in IMS and paves the way for positive proposals on what future research can do to fill the gaps in the existing literature, specifically encompassing both the intra-personal and inter-personal foundations of IMS.

### 4.1 Heterogeneity of measures: deciphering what movements tell

Given the variability of IMS measures employed in the body of literature reviewed here, one may ask whether they quantify similar characteristics of IMS. For instance, some authors used continuous relative phase to compute (i) the percentage of in-phase segments (83), (ii) the average amount of time a dyad spends in a given relative phase (89) or (iii) the circular variance of relative phase (84). Some other authors computed motion energy across defined regions of interest and subsequent dyadic cross-correlations (56, 86, 90), and yet others have assessed synchrony by means of weighted coherence (85, 88). Many other measures have also been employed, such as the percentage and duration of co-confident periods (80), reaction times, coefficient of variation of reaction times, movement time and asynchrony of reaching (61), differences of response times (62, 63) and rates of synchronized tapping (87).

As suggested by Schoenherr et al. (95), the specific aspect of synchrony being measured relies on the algorithm employed and the resulting score utilized. Accordingly, the literature on the field of motor development indicates that different motor parameters are associated with distinct neural activities and influenced by various factors such as cognitive, metacognitive, sensory, and social processes (96–98). However, specific mappings between measurement methods and underlying aspects of IMS require further investigation. If we take acceleration – a motor parameter related to movement smoothness – as an example, we can see how different measures tap into its multiple facets. By looking at the rate of acceleration change over time (99, 100), reach-to-grasp movement smoothness is shown to be influenced by factors like the presence or absence of the target object, its orientation, and the plane of movement (101). In turns, neural measures could reveal that movement acceleration aligns with coherent activation of contralateral primary motor (M1) hand area and involves dorsolateral prefrontal cortex (DLPFC) and posterior parietal cortex (PPC) for goal-directed action planning and sensorimotor integration/movement monitoring (102). Such accurate mapping is far from being available in relation to IMS. Although there is currently a lack of comprehensive literature elucidating the exact meaning of each measure in relation to different aspects of IMS, it is crucial to exercise caution when combining different measures. Without a clear understanding of which specific aspects of IMS each measurement or task taps into, the interpretation and comparison of effect sizes across studies can be challenging. To ensure meaningful comparisons, future research should aim to bridge this gap in knowledge by investigating the relationships between specific measurement methods, the underlying aspects of IMS they capture, and the resulting variation in observed effect sizes.

### 4.2 Context matters: towards naturalistic interactions

The experimental context might also modulate individuals’ ability to synchronize with others. As the systematic review showed, researchers relied on a variety of tasks that span from ecological and spontaneous activities to more controlled experimental contexts. One could argue that the uncertainty linked to the effect size may relate to the extent to which the interaction under consideration was ecological. For example, the study with the largest effect was also the study using the least structured paradigm (i.e., shared reading activity; 56). Remarkably enough, this study turns out to be the most influential according to sensitivity analyses to the extent that removing it from the analyses would importantly reduce the between-study heterogeneity, while the effect size would remain large. Such a scenario supports the idea that the type of task is indeed what makes this study different from others, although the phenomena it measures is the same, and that taking the context into account could also be key in the study of differences in the ability to synchronize with others. While the large effect size found by Liu et al. (56) might suggest an overestimation of the true effect, it could also be that we are dealing with a general underestimation of the effect size by the other studies due to a more controlled experimental setting that perhaps does not always capture the complexity of human exchanges. As highlighted by multiple authors, synchrony also depends on task demands and the context in which it is elicited (3, 60, 79).

Since individuals on the autism spectrum seem less sensitive to changes in the social context while aligning with others (3), they could possibly benefit from controlled settings while experiencing enhanced difficulties in a daily life context. If this was the case, controlled contexts might be an important arena to train IMS especially at early developmental stages, with a view of progressively expanding to less structured and more ecological situations in which possible cascading effects on higher-order social skills could be observed. Indeed, reduced IMS has been suggested as a proxy for social difficulties observed in autism, while the alignment of bodies has been shown to enhance social exchanges (103). On a continuum from more naturalistic to controlled setting, one could also suggest exploring differences between synchronizing with real vs virtual partners. In fact, literature shows differences between human and non-human or digital interactions. For instance, social interactions in VR come with less nuanced non-verbal cues. This has been shown to lead to changed regulation of interpersonal behaviors in neurotypical individuals but not in people on the autism spectrum (57). Exploring motor synchronization abilities of individuals within the autism spectrum with non-social or less social partners, such as avatars or digital characters (53, 55) holds relevance for gaining understanding of human interactions, particularly in light of potential differences in IMS associated with the double-empathy problem, as well as on how to enhance motor synchronization abilities with cascading effects on socio-cognitive outcomes.

The extent to which experimental settings reflect naturalistic interactions may also be reflected in the type of synchrony elicited. We did not find any effect of type of synchrony in our moderation analyses nor conflicting results in the systematic review, but this might be due to the low number of studies available. Whether the spontaneous vs induced IMS modulate the effect under investigation remains therefore an open question. As Howard et al. (79) interestingly highlighted, instructed synchrony could be particularly beneficial for children. In their study, they showed that although social bonding remained consistent across both groups irrespective of synchrony type, a heightened sense of social closeness correlated positively with increased synchronization accuracy in children only (79). While the limited sample of the current review and meta-analysis did not allow to explore any effect of age, exploring the interaction between the type of synchrony elicited and participants’ age in the context of autism and typical development could constitute an interesting avenue to understand the origins of diversity. Participants’ age in the included studies spans from 2 to 51 years and analyzing possible developmental changes would provide crucial insights on the reasons behind diversity across the lifespan and in ASD.

### 4.3 Beyond the individual: an interactive, inter-personal account of IMS in autism

Most studies have only investigated IMS in ASD-TD vs TD-TD dyads, with an implicit assumption of diversity in ASD compared to TD. Indeed, reduced synchrony in ASD-TD dyads is almost always attributed to intra-individual characteristics of autism and to the autistic member of the interaction. For example, symptom severity seems negatively correlated with IMS, in turns predictive of improved social cognition (85, 104). As autistic traits increase in neurotypical adults, both spontaneous (49) and induced IMS decrease and this appears to be modulated by motor difficulties (51), as previously found in ASD population (80). This well-established approach fails to consider that the neurotypical member of the dyad might itself face challenges in synchronizing with the neurodiverse member. In line, a growing number of researchers and members of the autism community emphasizes how deficit-based research on autism neglects that misattunement between individuals with and without ASD is bidirectional and multifaceted, with difficulties in interactions coming from differences in experiencing the world, rather than autistic deficits (45, 105).

In attempting to explain what may be the reasons that lead to impaired motor synchrony in ASD-TD dyads, it is crucial to consider that it is not necessarily the ASD member that has to some extent an impairment in the ability to synchronize, but that the TD member itself may have difficulty synchronizing with the ASD member because there are differences in sensorimotor and cognitive functioning that do not facilitate decoding the signals that the other is sending, as if each is tuned to different communication frequencies, or speaks different languages. In other words, we suggest that individuals tend to synchronize more with those who enable them to make more accurate predictions more easily.

For example, Noel et al. (90) showed diversity in levels of motor complexity (stereotyping, rhythmicity, predictability) in individuals in the spectrum or neurotypicals, and although the authors did not find a correlation between this factor and levels of motor synchrony at the dyadic level, it is possible that similarity in this parameter contributes to better synchronization. This means that if there is consistency in the level of motor complexity of the two interacting individuals, they will be able to make more accurate predictions and thus synchronize more easily. To put it simpler, imagine two people trying to have a conversation, but they speak different languages. It would be challenging for them to understand each other and find common ground. Similarly, when two individuals with different sensorimotor, cognitive, socio-emotional, and relational characteristics interact, there can be a significant gap between their understanding of each other. The same logic can be applied to sensory as well as motor functioning, that is, if there is similarity in the perception of multisensory cues from the external (as well as internal) world of the interacting individuals, it will be more immediate to synchronize because the decoding of the multisensory cues sent by the other will be easier, somewhat like speaking the same language. The more the interactive rhythm can be grasped and decoded similarly among the individuals who are interacting as a shared communication channel, the more they will manifest high levels of synchrony. Similarities in sensorimotor functioning contribute to a form of ingroup which, to note, does not necessarily correspond with a diagnostic label but rather with a constellation of functional modalities (106).

Even though reduced IMS has been also found in ASD-ASD dyads (86), deeper investigations would shed light on whether sharing similar experiences of the world support synchronization with one another. This hypothesis is supported by preliminary evidence provided by Glass and Yuill (60) in their recent work. One could however argue that the ability to synchronize with others is inherently related to one’s social skills. In the present work we have not been able to include social skills as a moderator due to the limited data available; however future research should carefully consider this factor. Indeed, independently from being on the autism spectrum or not, individual differences in social skills might relate to differences in the ability to synchronize with others, with possible bidirectional influences. This perspective would be particularly relevant to gain insights on intra-personal characteristics contributing to IMS regardless of neurodevelopmental conditions.

Embracing a Bayesian view, the process of synchronization with each other can be viewed as a nonlinear probabilistic combination of social and internal cues, and by the principle of resource optimization, the individual will tend to align more closely with those who allow him or her to make more accurate predictions more easily (39, 40). The concept of similarity (inter-individual level) in functional characteristics (intra-individual level, i.e., sensorimotor, cognitive, socio-emotional functioning) between two interacting individuals facilitates a more accurate prediction of each other’s intentions and actions, promoting a smoother synchronization process. The commonality in internal models and representations could in fact contribute to greater accuracy of individuals’ prior, with increasingly less need to update the predictive model in light of new information. In other words, by reducing the initial self-other gap, similarity enables more accurate priors and a more efficient convergence of the predictive model that in turns scaffolds the synchronization process.

## 5 Conclusions and future directions

Both our systematic review and meta-analysis shows that ASD-TD dyads, compared to TD-TD dyads, manifest reduced synchronization of their behaviors during social interactions, although high uncertainty emerged as for the true effect size. Although it is reasonable to believe that many intra-personal (e.g., age, sensorimotor, social, and cognitive profiles) and inter-personal factors (e.g., type of synchrony, similarity between the dyad members) may contribute to reduced IMS found in ASD-TD exchanges, from the available literature it is not possible to draw conclusive inferences because they are hardly accounted for.

To our knowledge, this is the first meta-analytic study on the topic and crucially contributes to the field providing a first effort towards a quantitative synthesis, but the results should be taken with caution given the limitations herein the available literature (i.e., few studies, diverse samples, various measures, theoretical bias in favor of deficit-based accounts of autism). The conduction of multi-site studies appears a feasible solution to solve the disparities across studies that emerged with the present work, as the use of common protocols and measures would allow a more precise evaluation of the phenomena of interest, especially including more consistent and focused age ranges that would the investigation of its developmental trajectories. Importantly, the current work paves the way for future research exploring IMS in ASD social interactions, which could improve perceived social connection and well-being.

## 6 Supplementary Information

The codes to reproduce the analyses reported in this article and in the SM have been made publicly available via the Open Science Framework (OSF) at the following URL: https://osf.io/dqjyh/.

SM include statistical analyses performed with *r* = .30 and *r* = .70. In addition, the online repository includes: R directory to run the analyses, raw dataset for analyses, dataset including information of each selected study, dataset used to compile the PRISMA flow diagram and plain R code syntax to reproduce the analysis results reported in the paper.

## Data availability statement

The original contributions presented in the study are included in the article/**Supplementary Material**. Further inquiries can be directed to the corresponding author.

## Author contributions

LC: Conceptualization, Data curation, Formal Analysis, Investigation, Methodology, Visualization, Writing – original draft. IV: Conceptualization, Data curation, Investigation, Methodology, Writing – original draft. GM: Data curation, Investigation, Writing – review & editing. GA: Formal Analysis, Methodology, Supervision, Writing – review & editing. TF: Conceptualization, Funding acquisition, Methodology, Supervision, Writing – review & editing.
