# Supplementary material for: Interpersonal motor synchrony in autism: a systematic review and meta-analysis
Source: Front Psychiatry. 2024 Feb 19;15:1355068. doi: 10.3389/fpsyt.2024.1355068 (PMC10909819; doi:10.3389/fpsyt.2024.1355068)
Supplement: Supplementary file 1 [file DataSheet_1.pdf]

SUPPLEMENTARY MATERIALS

Contents

Meta-analyses with r = .30 3
Dataset . . . 3
Descriptive statistics . . . 3
Random-effects meta-analysis . . . 3
Models . . . 3
Forest plot . . . 4
Prediction interval . . . 5
Funnel plot (trim-and-fill method) . . . 5
Model corrected for publication bias (trim-and-fill method) . . . 6
Sensitivity analyses . . . 6
Leave-One-Out . . . 6
Cook's distance . . . 6

Meta-analyses with r = .50 8
Dataset . . . 8
Descriptive statistics . . . 8
Random-effects meta-analysis . . . 8
Models . . . 8
Forest plot . . . 9
Funnel plot (trim-and-fill method) . . . 10
Model corrected for publication bias (trim-and-fill method) . . . 11
Sensitivity analyses . . . 11
Leave-One-Out . . . 11
Cook's distance . . . 11

Meta-analyses with r = .70 13
Dataset . . . 13
Descriptive statistics . . . 13
Random-effects meta-analysis . . . 13
Models . . . 13

Forest plot . . . 14
Prediction interval . . . 15
Funnel plot (trim-and-fill method) . . . 15
Model corrected for publication bias (trim-and-fill method) . . . 16
Sensitivity analyses . . . 16
    Leave-One-Out . . . 16
    Cook's distance . . . 16

Comparing results **17**

Meta-analyses with r = .30

Dataset

Effect sizes (Hedge's g) and varinaces for each of the included studies, with $r = .30$

ID	Authors	Hedge's g	Var
1	Brezis et al., 2017	0.66	0.03
2	Fitzpatrick et al., 2013	0.21	0.25
3	Fitzpatrick et al., 2017	1.00	0.04
4	Fitzpatrick et al., 2016	0.34	0.09
5	Fulceri et al., 2018	0.88	0.08
6	Georgescu et al., 2020	0.83	0.08
7	Kawasaki et al., 2017	0.83	0.09
8	Kruppa et al., 2021	0.62	0.05
9	Lampi et al., 2020	0.83	0.03
10	Liu et al., 2021	3.78	0.10
11	Marsh et al., 2013	0.21	0.31
12	Noel et al., 2018	0.24	0.09
13	Yoo et al., 2018	0.44	0.05

Descriptive statistics

			TD Group					ASD Group							
					Age					Age					
ID	Authors	Country	N	M/F ratio	range	mean	sd	N	M/F ratio	range	mean	sd	Type of Synchrony	Hedge's g	var
1	Brezis et al., 2017	Israel	35	28:7	19 - 45	25.90	6.37	34	31:3	20 - 45	28.60	6.26	instructed	0.66	0.03
2	Fitzpatrick et al., 2013	USA	3	1:2	4 - 5.6	4.80	0.75	5	4:1	5 - 7.4	6.21	1.17	instructed	0.21	0.25
3	Fitzpatrick et al., 2017	USA	27	21:6	6.33 - 10.8	8.24	1.46	23	20:3	6.08 - 10.75	8.08	1.44	instructed	1.00	0.04
4	Fitzpatrick et al., 2016	USA	9	7:2	12 - 16	14.44	1.13	9	8:1	12 - 17	13.67	1.94	instructed	0.34	0.09
5	Fulceri et al., 2018	Italy	11	9:2	6.3 - 9.8	7.57	0.71	11	10:1	5.11 - 10.3	7.82	1.32	spontaneous	0.88	0.08
6	Georgescu et al., 2020	Germany	10	6:4	33 - 51	41.80	8.86	9	5:4	30 - 50	40.72	10.45	spontaneous	0.83	0.08
7	Kawasaki et al., 2017	USA	24	12:12	18.9 - 32.1	25.60	6.60	24	14:10	22 - 36.4	29.20	7.20	instructed	0.83	0.09
8	Kruppa et al., 2021	Germany	41	18:23	8 - 18	12.66	2.79	18	18:0	8 - 18	13.54	2.96	instructed	0.62	0.05
9	Lampi et al., 2020	USA	47	34:13	6 - 10	7.85	1.49	50	34:7	6 - 10	8.02	1.44	spontaneous	0.83	0.03
10	Liu et al., 2021	USA	16	10:6	1.66 - 4.33	2.99	0.70	13	10:3	1.75 - 5.75	3.88	0.85	spontaneous	3.78	0.10
11	Marsh et al., 2013	USA	7	4:3	2.8 - 4.6	3.75	0.12	7	5:2	3.8 - 4.1	3.94	0.74	spontaneous	0.21	0.31
12	Noel et al., 2018	USA	15	11:4	8.9 - 14.5	10.94	2.13	12	8:4	7.9 - 16.5	12.20	3.75	spontaneous	0.24	0.09
13	Yoo et al., 2018	Korea	42	23:19	11 - 16	13.50	0.80	10	10:0	11 - 16	13.40	1.40	spontaneous	0.44	0.05

Random-effects meta-analysis

Models

```
m.random <- rma(yi=es, vi=var, data=df_agg, method="REML")
RE.results <- summary(m.random)
print(RE.results)
```

```
##
## Random-Effects Model (k = 13; tau^2 estimator: REML)
##
##    logLik  deviance       AIC       BIC      AICc
## -16.0619   32.1238   36.1238   37.0936   37.4571
##
```

```
## tau^2 (estimated amount of total heterogeneity): 0.7439 (SE = 0.3402)
## tau (square root of estimated tau^2 value):      0.8625
## I^2 (total heterogeneity / total variability):   92.19%
## H^2 (total variability / sampling variability):  12.80
##
## Test for Heterogeneity:
## Q(df = 12) = 105.5515, p-val < .0001
##
## Model Results:
##
## estimate      se    zval    pval   ci.lb   ci.ub
##   0.8494  0.2535  3.3505  0.0008  0.3525  1.3462  ***
##
## ---
## Signif. codes:  0 '***' 0.001 '**' 0.01 '*' 0.05 '.' 0.1 ' ' 1
```

```
#fit moderation model (type of synchrony)
moderation.random <- rma(yi=es, vi=var, mods = ~ synch_type, data=df_agg, method="REML")
summary(moderation.random)
```

```
##
## Mixed-Effects Model (k = 13; tau^2 estimator: REML)
##
##   logLik  deviance       AIC       BIC      AICc
## -14.8883   29.7765   35.7765   36.9702   39.2051
##
## tau^2 (estimated amount of residual heterogeneity):     0.7701 (SE = 0.3669)
## tau (square root of estimated tau^2 value):             0.8776
## I^2 (residual heterogeneity / unaccounted variability): 92.14%
## H^2 (unaccounted variability / sampling variability):   12.73
## R^2 (amount of heterogeneity accounted for):            0.00%
##
## Test for Residual Heterogeneity:
## QE(df = 11) = 101.4966, p-val < .0001
##
## Test of Moderators (coefficient 2):
## QM(df = 1) = 0.6647, p-val = 0.4149
##
## Model Results:
##
##                        estimate      se    zval    pval    ci.lb   ci.ub
## intrcpt                  0.6241  0.3774  1.6535  0.0982  -0.1157  1.3638  .
## synch_typespontaneous    0.4208  0.5162  0.8153  0.4149  -0.5909  1.4326
##
## ---
## Signif. codes:  0 '***' 0.001 '**' 0.01 '*' 0.05 '.' 0.1 ' ' 1
```

Forest plot

Dotted line is the prediction interval

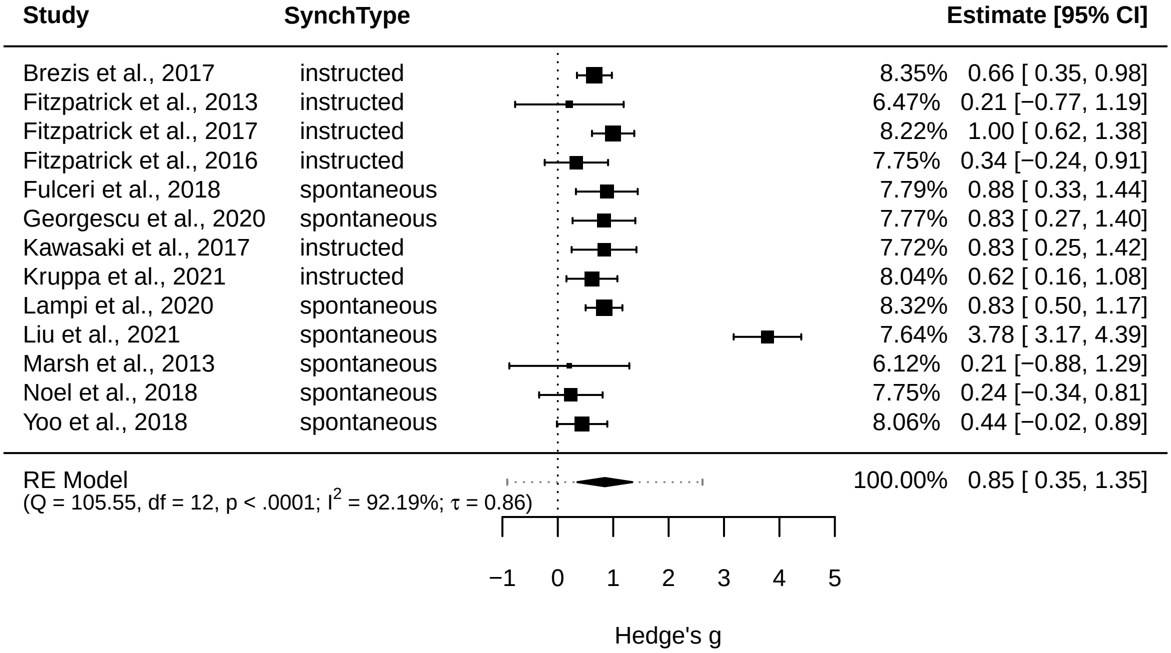

Prediction interval

```
##
##     pred     se  ci.lb  ci.ub   pi.lb  pi.ub
##   0.8494 0.2535 0.3525 1.3462 -0.9126 2.6113
```

Funnel plot (trim-and-fill method)

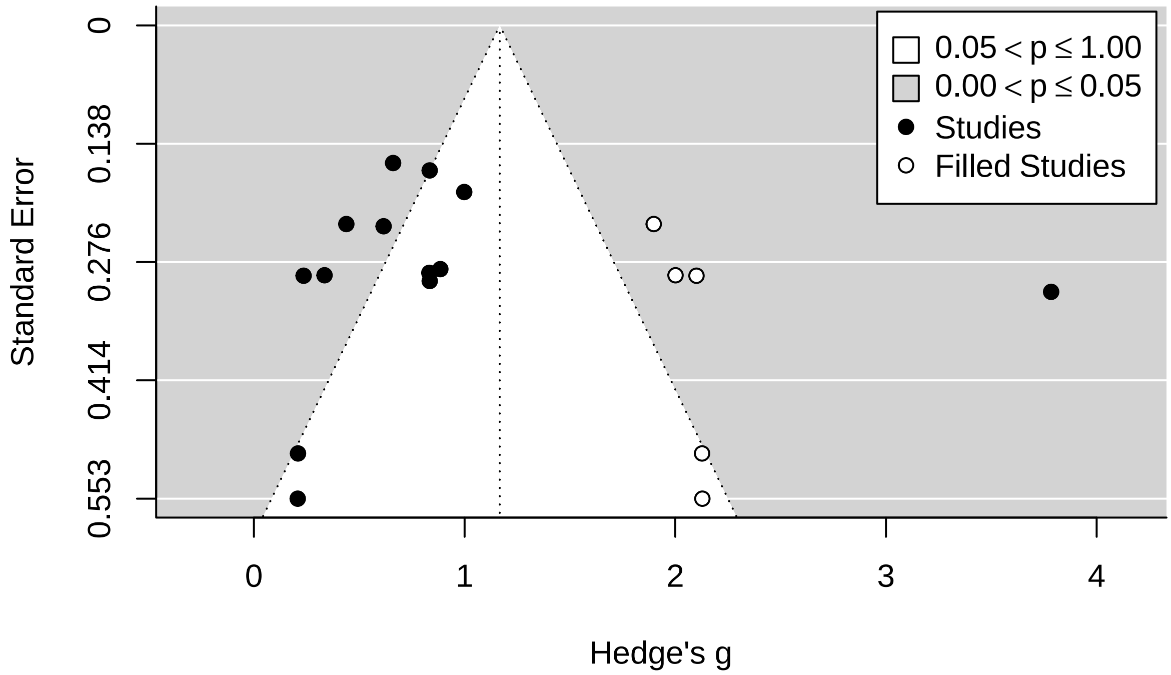

Model corrected for publication bias (trim-and-fill method)

```
##
## Estimated number of missing studies on the right side: 5 (SE = 2.2785)
##
## Random-Effects Model (k = 18; tau^2 estimator: REML)
##
##   logLik  deviance       AIC       BIC      AICc
## -23.3268   46.6537   50.6537   52.3201   51.5108
##
## tau^2 (estimated amount of total heterogeneity): 0.7886 (SE = 0.3059)
## tau (square root of estimated tau^2 value):      0.8881
## I^2 (total heterogeneity / total variability):   91.81%
## H^2 (total variability / sampling variability):  12.21
##
## Test for Heterogeneity:
## Q(df = 17) = 160.8662, p-val < .0001
##
## Model Results:
##
## estimate      se    zval    pval   ci.lb   ci.ub
##   1.1668  0.2230  5.2335  <.0001  0.7299  1.6038  ***
##
## ---
## Signif. codes:  0 '***' 0.001 '**' 0.01 '*' 0.05 '.' 0.1 ' ' 1
```

Sensitivity analyses

Authors	Estimate	I2	tau	CI	PI
Brezis et al., 2017	0.87	92.08	0.91	[0.32;1.41]	[-0.99;2.73]
Fitzpatrick et al., 2013	0.89	92.96	0.88	[0.37;1.42]	[-0.92;2.7]
Fitzpatrick et al., 2016	0.89	92.79	0.89	[0.36;1.42]	[-0.93;2.72]
Fitzpatrick et al., 2017	0.84	92.50	0.91	[0.29;1.38]	[-1.03;2.7]
Fulceri et al., 2018	0.85	93.00	0.91	[0.30;1.39]	[-1.01;2.7]
Georgescu et al., 2020	0.85	93.01	0.91	[0.31;1.39]	[-1.01;2.71]
Kawasaki et al., 2017	0.85	93.03	0.91	[0.31;1.39]	[-1.01;2.71]
Kruppa et al., 2021	0.87	92.75	0.91	[0.33;1.41]	[-0.99;2.72]
Lampi et al., 2020	0.85	92.24	0.91	[0.31;1.39]	[-1.02;2.71]
Liu et al., 2021	0.68	1.36	0.00	[0.54;0.82]	[0.53;0.83]
Marsh et al., 2013	0.89	92.98	0.88	[0.37;1.42]	[-0.92;2.7]
Noel et al., 2018	0.90	92.69	0.88	[0.37;1.43]	[-0.91;2.71]
Yoo et al., 2018	0.88	92.63	0.90	[0.35;1.42]	[-0.96;2.72]

Leave-One-Out

Cook's distance

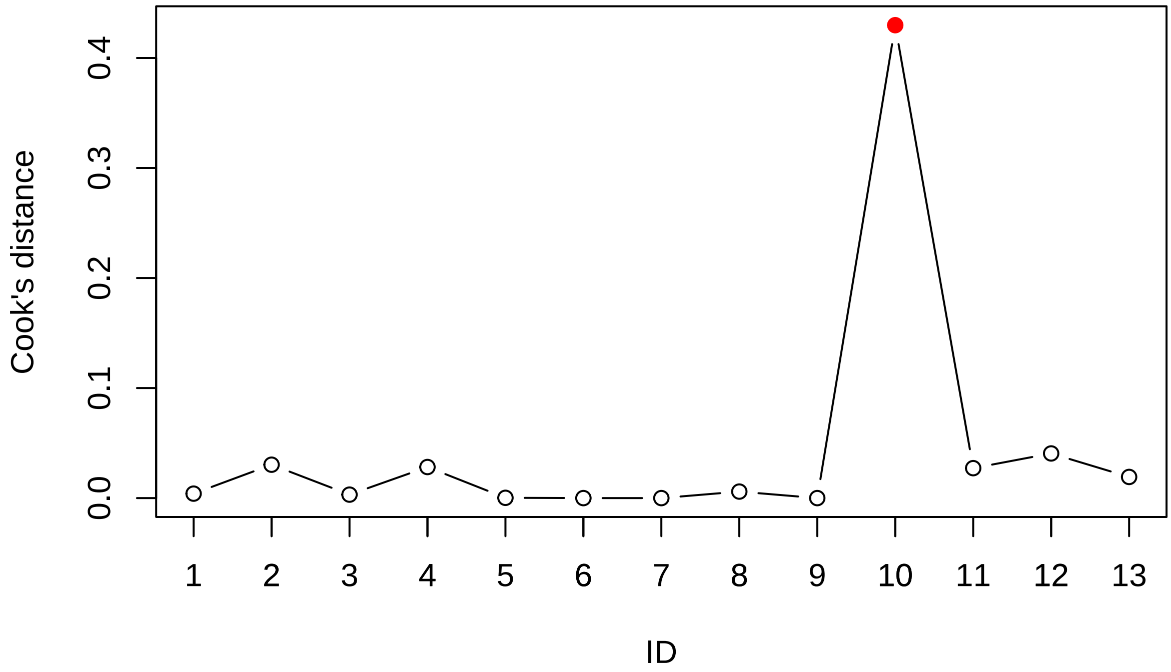

Note that study IDs follow alphabetical order of included studies and their specifications reported in the descriptive statistic's table

Meta-analyses with r = .50

Dataset

Effect sizes (Hedge's g) and varinaces for each of the included studies, with $r = .50$

ID	Authors	Hedge's g	Var
1	Brezis et al., 2017	0.66	0.04
2	Fitzpatrick et al., 2013	0.21	0.34
3	Fitzpatrick et al., 2017	1.00	0.05
4	Fitzpatrick et al., 2016	0.34	0.13
5	Fulceri et al., 2018	0.88	0.11
6	Georgescu et al., 2020	0.83	0.12
7	Kawasaki et al., 2017	0.83	0.09
8	Kruppa et al., 2021	0.62	0.06
9	Lampi et al., 2020	0.83	0.03
10	Liu et al., 2021	3.78	0.11
11	Marsh et al., 2013	0.21	0.31
12	Noel et al., 2018	0.24	0.11
13	Yoo et al., 2018	0.44	0.08

Descriptive statistics

			TD Group					ASD Group							
					Age					Age					
ID	Authors	Country	N	M/F ratio	range	mean	sd	N	M/F ratio	range	mean	sd	Type of Synchrony	Hedge's g	var
1	Brezis et al., 2017	Israel	35	28:7	19 - 45	25.90	6.37	34	31:3	20 - 45	28.60	6.26	instructed	0.66	0.04
2	Fitzpatrick et al., 2013	USA	3	1:2	4 - 5.6	4.80	0.75	5	4:1	5 - 7.4	6.21	1.17	instructed	0.21	0.34
3	Fitzpatrick et al., 2017	USA	27	21:6	6.33 - 10.8	8.24	1.46	23	20:3	6.08 - 10.75	8.08	1.44	instructed	1.00	0.05
4	Fitzpatrick et al., 2016	USA	9	7:2	12 - 16	14.44	1.13	9	8:1	12 - 17	13.67	1.94	instructed	0.34	0.13
5	Fulceri et al., 2018	Italy	11	9:2	6.3 - 9.8	7.57	0.71	11	10:1	5.11 - 10.3	7.82	1.32	spontaneous	0.88	0.11
6	Georgescu et al., 2020	Germany	10	6:4	33 - 51	41.80	8.86	9	5:4	30 - 50	40.72	10.45	spontaneous	0.83	0.12
7	Kawasaki et al., 2017	USA	24	12:12	18.9 - 32.1	25.60	6.60	24	14:10	22 - 36.4	29.20	7.20	instructed	0.83	0.09
8	Kruppa et al., 2021	Germany	41	18:23	8 - 18	12.66	2.79	18	18:0	8 - 18	13.54	2.96	instructed	0.62	0.06
9	Lampi et al., 2020	USA	47	34:13	6 - 10	7.85	1.49	50	34:7	6 - 10	8.02	1.44	spontaneous	0.83	0.03
10	Liu et al., 2021	USA	16	10:6	1.66 - 4.33	2.99	0.70	13	10:3	1.75 - 5.75	3.88	0.85	spontaneous	3.78	0.11
11	Marsh et al., 2013	USA	7	4:3	2.8 - 4.6	3.75	0.12	7	5:2	3.8 - 4.1	3.94	0.74	spontaneous	0.21	0.31
12	Noel et al., 2018	USA	15	11:4	8.9 - 14.5	10.94	2.13	12	8:4	7.9 - 16.5	12.20	3.75	spontaneous	0.24	0.11
13	Yoo et al., 2018	Korea	42	23:19	11 - 16	13.50	0.80	10	10:0	11 - 16	13.40	1.40	spontaneous	0.44	0.08

Random-effects meta-analysis

Models

```
m.random <- rma(yi=es, vi=var, data=df_agg, method="REML")
RE.results <- summary(m.random)
print(RE.results)
```

```
##
## Random-Effects Model (k = 13; tau^2 estimator: REML)
##
##    logLik  deviance       AIC       BIC      AICc
## -16.0955   32.1909   36.1909   37.1607   37.5242
##
```

```
## tau^2 (estimated amount of total heterogeneity): 0.7307 (SE = 0.3433)
## tau (square root of estimated tau^2 value):      0.8548
## I^2 (total heterogeneity / total variability):   90.06%
## H^2 (total variability / sampling variability):  10.06
##
## Test for Heterogeneity:
## Q(df = 12) = 89.7806, p-val < .0001
##
## Model Results:
##
## estimate      se    zval    pval   ci.lb   ci.ub
##   0.8534  0.2547  3.3500  0.0008  0.3541  1.3527  ***
##
## ---
## Signif. codes:  0 '***' 0.001 '**' 0.01 '*' 0.05 '.' 0.1 ' ' 1
```

```r
#fit moderation model (type of synchrony)
moderation.random <- rma(yi=es, vi=var, mods = ~ synch_type, data=df_agg, method="REML")
summary(moderation.random)
```

```
##
## Mixed-Effects Model (k = 13; tau^2 estimator: REML)
##
##   logLik  deviance       AIC       BIC      AICc
## -14.9281   29.8562   35.8562   37.0499   39.2848
##
## tau^2 (estimated amount of residual heterogeneity):     0.7596 (SE = 0.3714)
## tau (square root of estimated tau^2 value):             0.8716
## I^2 (residual heterogeneity / unaccounted variability): 90.03%
## H^2 (unaccounted variability / sampling variability):   10.03
## R^2 (amount of heterogeneity accounted for):            0.00%
##
## Test for Residual Heterogeneity:
## QE(df = 11) = 86.0983, p-val < .0001
##
## Test of Moderators (coefficient 2):
## QM(df = 1) = 0.6403, p-val = 0.4236
##
## Model Results:
##
##                        estimate      se    zval    pval    ci.lb   ci.ub
## intrcpt                  0.6303  0.3802  1.6579  0.0973  -0.1149  1.3756  .
## synch_typespontaneous    0.4157  0.5195  0.8002  0.4236  -0.6026  1.4340
##
## ---
## Signif. codes:  0 '***' 0.001 '**' 0.01 '*' 0.05 '.' 0.1 ' ' 1
```

Forest plot

Dotted line is the prediction interval

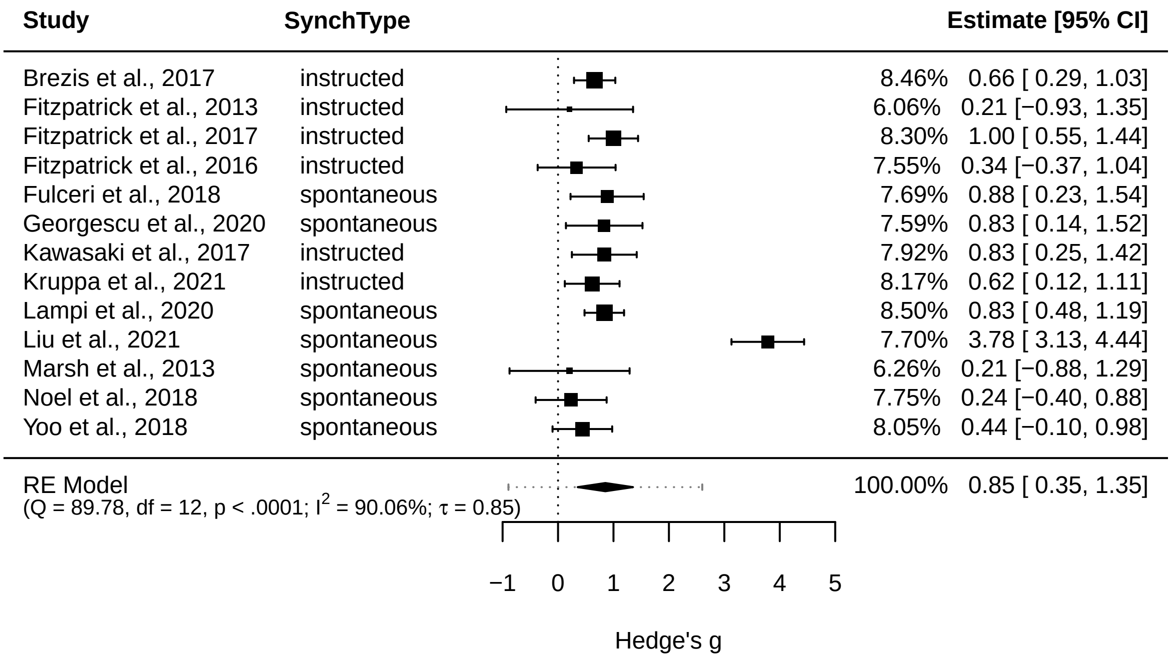

Prediction interval

```
##
##    pred     se  ci.lb  ci.ub   pi.lb  pi.ub
##  0.8534 0.2547 0.3541 1.3527 -0.8948 2.6017
```

Funnel plot (trim-and-fill method)

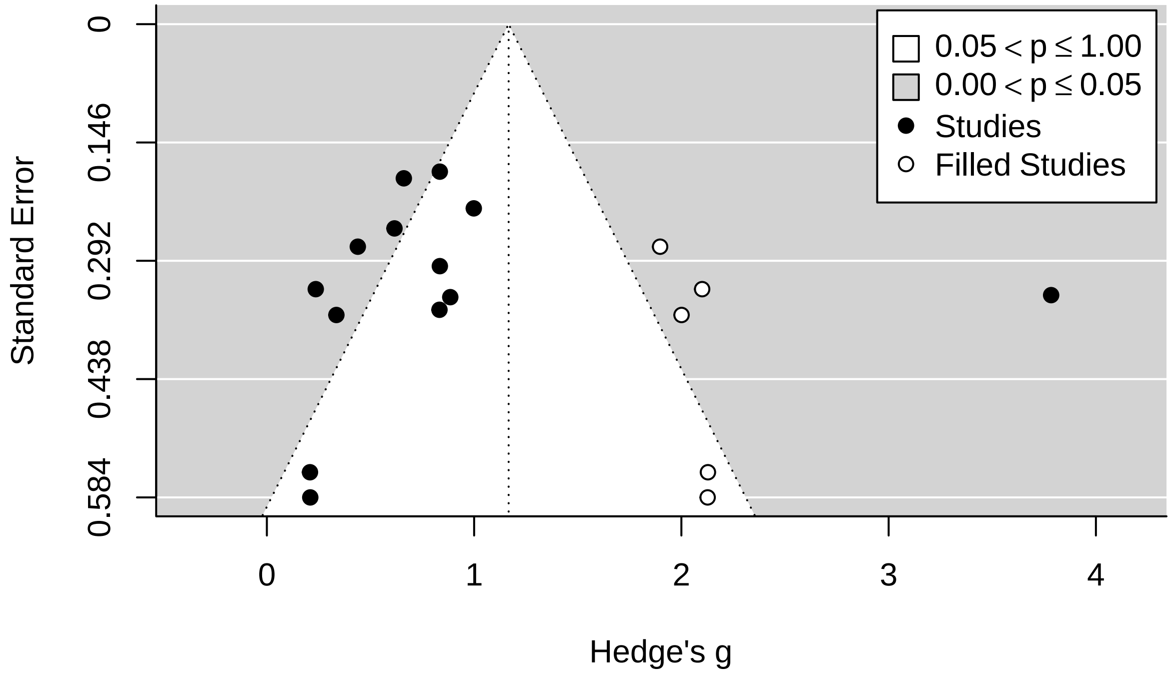

Model corrected for publication bias (trim-and-fill method)

```
##
## Estimated number of missing studies on the right side: 5 (SE = 2.2785)
##
## Random-Effects Model (k = 18; tau^2 estimator: REML)
##
##   logLik  deviance       AIC       BIC      AICc
## -23.3410   46.6819   50.6819   52.3483   51.5390
##
## tau^2 (estimated amount of total heterogeneity): 0.7668 (SE = 0.3065)
## tau (square root of estimated tau^2 value):      0.8757
## I^2 (total heterogeneity / total variability):   89.40%
## H^2 (total variability / sampling variability):  9.43
##
## Test for Heterogeneity:
## Q(df = 17) = 130.3017, p-val < .0001
##
## Model Results:
##
## estimate      se    zval    pval   ci.lb   ci.ub
##   1.1665  0.2233  5.2240  <.0001  0.7288  1.6041  ***
##
## ---
## Signif. codes:  0 '***' 0.001 '**' 0.01 '*' 0.05 '.' 0.1 ' ' 1
```

Sensitivity analyses

Authors	Estimate	I2	tau	CI	PI
Brezis et al., 2017	0.87	90.08	0.90	[0.32;1.42]	[-0.98;2.72]
Fitzpatrick et al., 2013	0.89	91.03	0.88	[0.37;1.42]	[-0.9;2.69]
Fitzpatrick et al., 2016	0.90	90.89	0.88	[0.36;1.43]	[-0.92;2.71]
Fitzpatrick et al., 2017	0.84	90.54	0.90	[0.29;1.39]	[-1.01;2.69]
Fulceri et al., 2018	0.85	91.12	0.90	[0.31;1.39]	[-1;2.69]
Georgescu et al., 2020	0.85	91.15	0.90	[0.31;1.40]	[-0.99;2.7]
Kawasaki et al., 2017	0.85	91.00	0.90	[0.31;1.40]	[-0.99;2.7]
Kruppa et al., 2021	0.87	90.69	0.90	[0.33;1.42]	[-0.97;2.72]
Lampi et al., 2020	0.85	90.00	0.91	[0.31;1.40]	[-1;2.71]
Liu et al., 2021	0.69	0.00	0.00	[0.53;0.84]	[0.53;0.84]
Marsh et al., 2013	0.90	91.01	0.88	[0.37;1.42]	[-0.9;2.69]
Noel et al., 2018	0.90	90.67	0.88	[0.37;1.44]	[-0.89;2.7]
Yoo et al., 2018	0.89	90.68	0.89	[0.35;1.43]	[-0.94;2.72]

Leave-One-Out

Cook's distance

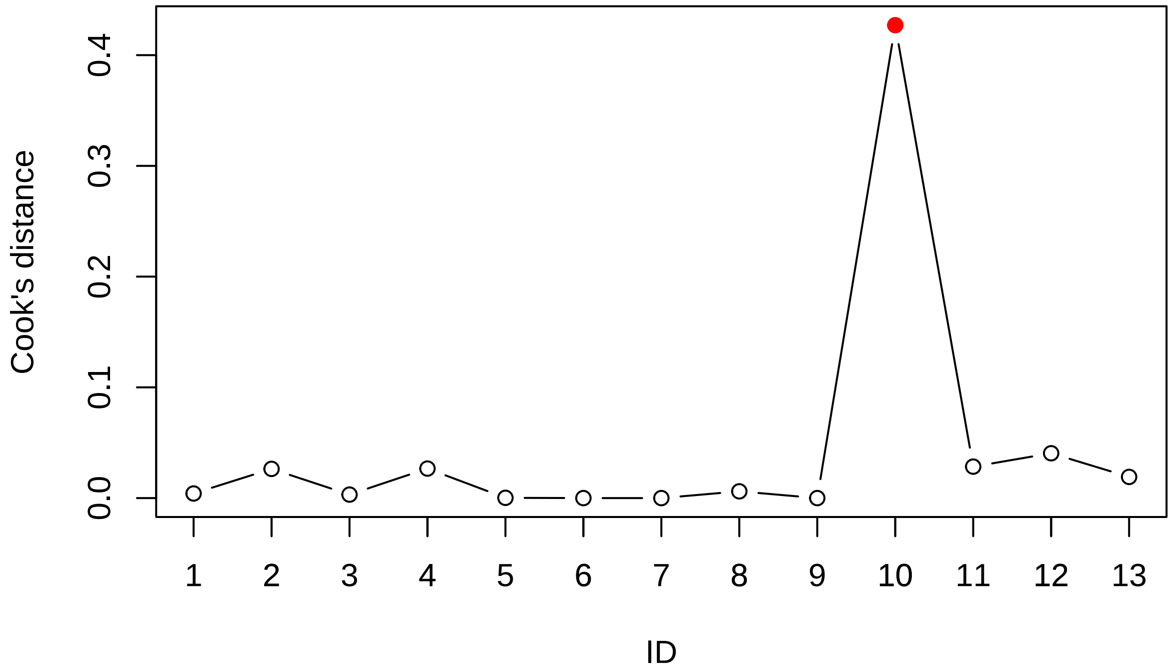

Note that study IDs follow alphabetical order of included studies and their specifications reported in the descriptive statistic's table

Meta-analyses with r = .70

Dataset

Effect sizes (Hedge's g) and varinaces for each of the included studies, with $r = .70$

ID	Authors	Hedge's g	Var
1	Brezis et al., 2017	0.66	0.05
2	Fitzpatrick et al., 2013	0.21	0.43
3	Fitzpatrick et al., 2017	1.00	0.07
4	Fitzpatrick et al., 2016	0.34	0.17
5	Fulceri et al., 2018	0.88	0.15
6	Georgescu et al., 2020	0.83	0.16
7	Kawasaki et al., 2017	0.83	0.09
8	Kruppa et al., 2021	0.62	0.07
9	Lampi et al., 2020	0.83	0.04
10	Liu et al., 2021	3.78	0.13
11	Marsh et al., 2013	0.21	0.31
12	Noel et al., 2018	0.24	0.13
13	Yoo et al., 2018	0.44	0.10

Descriptive statistics

			TD Group					ASD Group							
					Age					Age					
ID	Authors	Country	N	M/F ratio	range	mean	sd	N	M/F ratio	range	mean	sd	Type of Synchrony	Hedge's g	var
1	Brezis et al., 2017	Israel	35	28:7	19 - 45	25.90	6.37	34	31:3	20 - 45	28.60	6.26	instructed	0.66	0.05
2	Fitzpatrick et al., 2013	USA	3	1:2	4 - 5.6	4.80	0.75	5	4:1	5 - 7.4	6.21	1.17	instructed	0.21	0.43
3	Fitzpatrick et al., 2017	USA	27	21:6	6.33 - 10.8	8.24	1.46	23	20:3	6.08 - 10.75	8.08	1.44	instructed	1.00	0.07
4	Fitzpatrick et al., 2016	USA	9	7:2	12 - 16	14.44	1.13	9	8:1	12 - 17	13.67	1.94	instructed	0.34	0.17
5	Fulceri et al., 2018	Italy	11	9:2	6.3 - 9.8	7.57	0.71	11	10:1	5.11 - 10.3	7.82	1.32	spontaneous	0.88	0.15
6	Georgescu et al., 2020	Germany	10	6:4	33 - 51	41.80	8.86	9	5:4	30 - 50	40.72	10.45	spontaneous	0.83	0.16
7	Kawasaki et al., 2017	USA	24	12:12	18.9 - 32.1	25.60	6.60	24	14:10	22 - 36.4	29.20	7.20	instructed	0.83	0.09
8	Kruppa et al., 2021	Germany	41	18:23	8 - 18	12.66	2.79	18	18:0	8 - 18	13.54	2.96	instructed	0.62	0.07
9	Lampi et al., 2020	USA	47	34:13	6 - 10	7.85	1.49	50	34:7	6 - 10	8.02	1.44	spontaneous	0.83	0.04
10	Liu et al., 2021	USA	16	10:6	1.66 - 4.33	2.99	0.70	13	10:3	1.75 - 5.75	3.88	0.85	spontaneous	3.78	0.13
11	Marsh et al., 2013	USA	7	4:3	2.8 - 4.6	3.75	0.12	7	5:2	3.8 - 4.1	3.94	0.74	spontaneous	0.21	0.31
12	Noel et al., 2018	USA	15	11:4	8.9 - 14.5	10.94	2.13	12	8:4	7.9 - 16.5	12.20	3.75	spontaneous	0.24	0.13
13	Yoo et al., 2018	Korea	42	23:19	11 - 16	13.50	0.80	10	10:0	11 - 16	13.40	1.40	spontaneous	0.44	0.10

Random-effects meta-analysis

Models

```
m.random <- rma(yi=es, vi=var, data=df_agg, method="REML")
RE.results <- summary(m.random)
print(RE.results)
```

```
##
## Random-Effects Model (k = 13; tau^2 estimator: REML)
##
##    logLik  deviance       AIC       BIC      AICc
## -16.1275   32.2550   36.2550   37.2248   37.5883
##
```

```
## tau^2 (estimated amount of total heterogeneity): 0.7175 (SE = 0.3459)
## tau (square root of estimated tau^2 value):      0.8470
## I^2 (total heterogeneity / total variability):   88.11%
## H^2 (total variability / sampling variability):  8.41
##
## Test for Heterogeneity:
## Q(df = 12) = 78.4058, p-val < .0001
##
## Model Results:
##
## estimate      se    zval    pval   ci.lb   ci.ub
##   0.8570  0.2558  3.3498  0.0008  0.3556  1.3585  ***
##
## ---
## Signif. codes:  0 '***' 0.001 '**' 0.01 '*' 0.05 '.' 0.1 ' ' 1
```

```
#fit moderation model (type of synchrony)
moderation.random <- rma(yi=es, vi=var, mods = ~ synch_type, data=df_agg, method="REML")
summary(moderation.random)
```

```
##
## Mixed-Effects Model (k = 13; tau^2 estimator: REML)
##
##   logLik  deviance       AIC       BIC      AICc
## -14.9658   29.9316   35.9316   37.1253   39.3602
##
## tau^2 (estimated amount of residual heterogeneity):     0.7487 (SE = 0.3754)
## tau (square root of estimated tau^2 value):             0.8653
## I^2 (residual heterogeneity / unaccounted variability): 88.11%
## H^2 (unaccounted variability / sampling variability):   8.41
## R^2 (amount of heterogeneity accounted for):            0.00%
##
## Test for Residual Heterogeneity:
## QE(df = 11) = 75.0846, p-val < .0001
##
## Test of Moderators (coefficient 2):
## QM(df = 1) = 0.6188, p-val = 0.4315
##
## Model Results:
##
##                        estimate      se    zval    pval    ci.lb   ci.ub
## intrcpt                  0.6361  0.3826  1.6625  0.0964  -0.1138  1.3860  .
## synch_typespontaneous    0.4110  0.5225  0.7866  0.4315  -0.6130  1.4350
##
## ---
## Signif. codes:  0 '***' 0.001 '**' 0.01 '*' 0.05 '.' 0.1 ' ' 1
```

Forest plot

Dotted line is the prediction interval

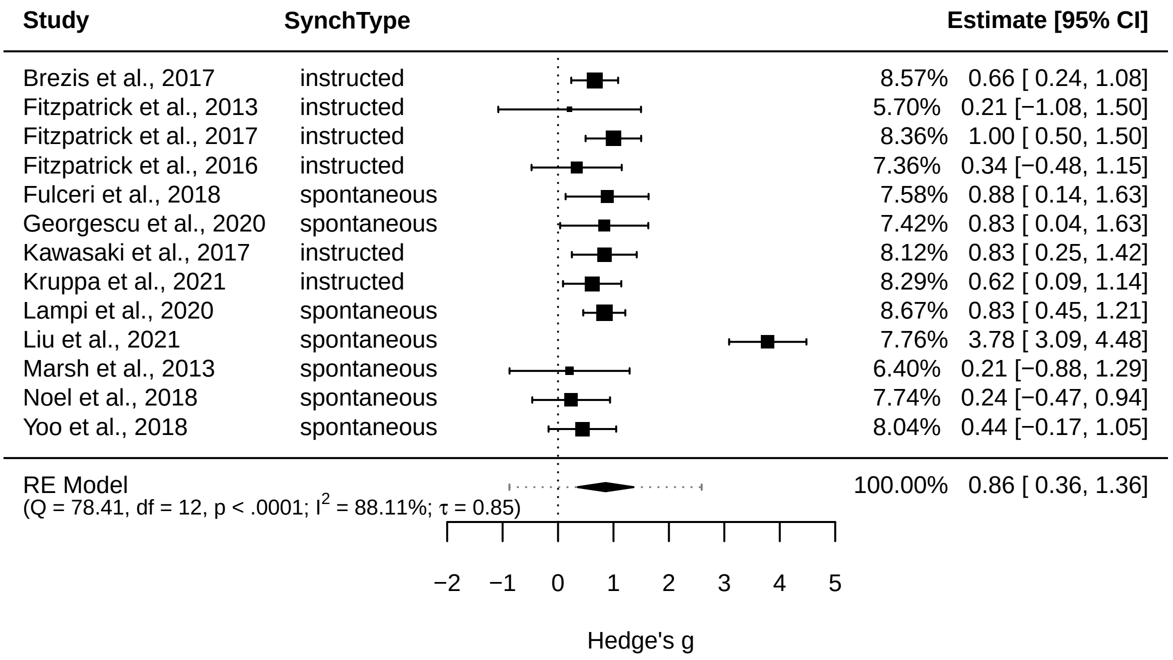

Prediction interval

```
##
##    pred     se  ci.lb  ci.ub   pi.lb  pi.ub
##  0.8570 0.2558 0.3556 1.3585 -0.8772 2.5913
```

Funnel plot (trim-and-fill method)

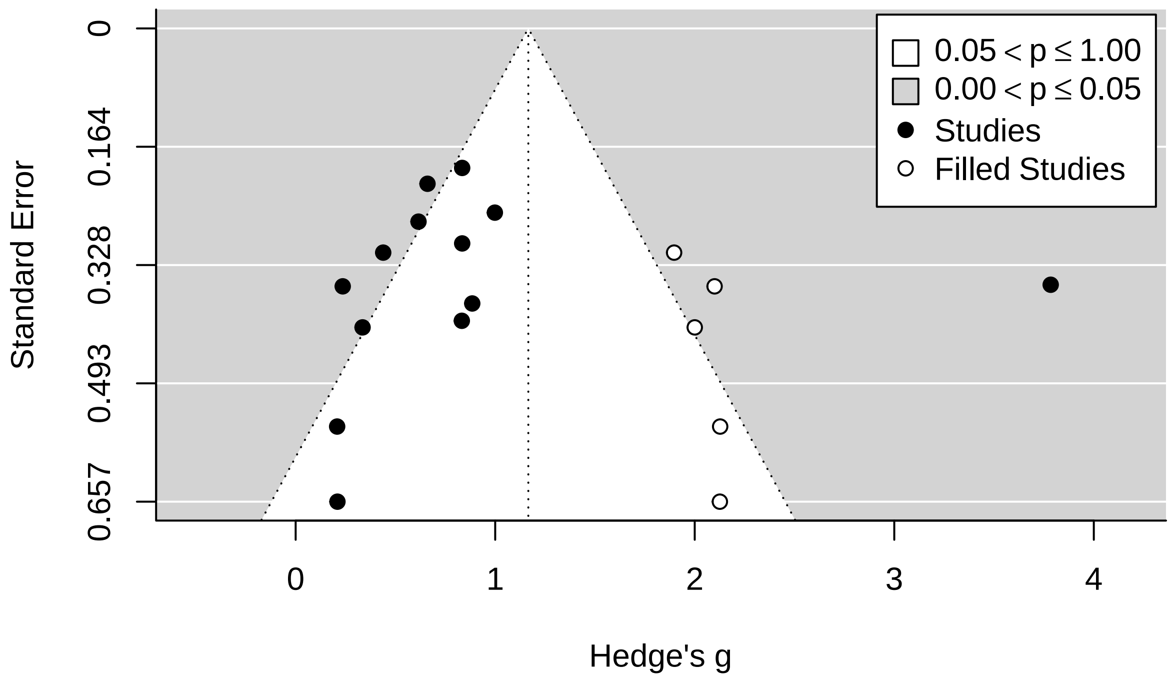

Model corrected for publication bias (trim-and-fill method)

```
##
## Estimated number of missing studies on the right side: 5 (SE = 2.2785)
##
## Random-Effects Model (k = 18; tau^2 estimator: REML)
##
##   logLik  deviance       AIC       BIC      AICc
## -23.3577   46.7154   50.7154   52.3819   51.5726
##
## tau^2 (estimated amount of total heterogeneity): 0.7463 (SE = 0.3072)
## tau (square root of estimated tau^2 value):      0.8639
## I^2 (total heterogeneity / total variability):   87.17%
## H^2 (total variability / sampling variability):  7.80
##
## Test for Heterogeneity:
## Q(df = 17) = 110.8582, p-val < .0001
##
## Model Results:
##
## estimate      se    zval    pval   ci.lb   ci.ub
##   1.1660  0.2237  5.2132  <.0001  0.7276  1.6044  ***
##
## ---
## Signif. codes:  0 '***' 0.001 '**' 0.01 '*' 0.05 '.' 0.1 ' ' 1
```

Sensitivity analyses

Authors	Estimate	I2	tau	CI	PI
Brezis et al., 2017	0.87	88.27	0.89	[0.32;1.42]	[-0.96;2.71]
Fitzpatrick et al., 2013	0.90	89.26	0.87	[0.37;1.42]	[-0.88;2.68]
Fitzpatrick et al., 2016	0.90	89.14	0.88	[0.36;1.43]	[-0.9;2.7]
Fitzpatrick et al., 2017	0.84	88.77	0.89	[0.29;1.39]	[-0.99;2.68]
Fulceri et al., 2018	0.85	89.40	0.89	[0.31;1.40]	[-0.98;2.68]
Georgescu et al., 2020	0.86	89.44	0.89	[0.31;1.40]	[-0.97;2.69]
Kawasaki et al., 2017	0.86	89.10	0.89	[0.31;1.41]	[-0.98;2.69]
Kruppa et al., 2021	0.88	88.81	0.89	[0.33;1.42]	[-0.95;2.71]
Lampi et al., 2020	0.86	87.98	0.90	[0.31;1.41]	[-0.99;2.7]
Liu et al., 2021	0.69	0.00	0.00	[0.52;0.86]	[0.52;0.86]
Marsh et al., 2013	0.90	89.18	0.87	[0.37;1.43]	[-0.88;2.68]
Noel et al., 2018	0.91	88.83	0.87	[0.37;1.44]	[-0.88;2.69]
Yoo et al., 2018	0.89	88.91	0.88	[0.35;1.43]	[-0.92;2.71]

Leave-One-Out

Cook's distance

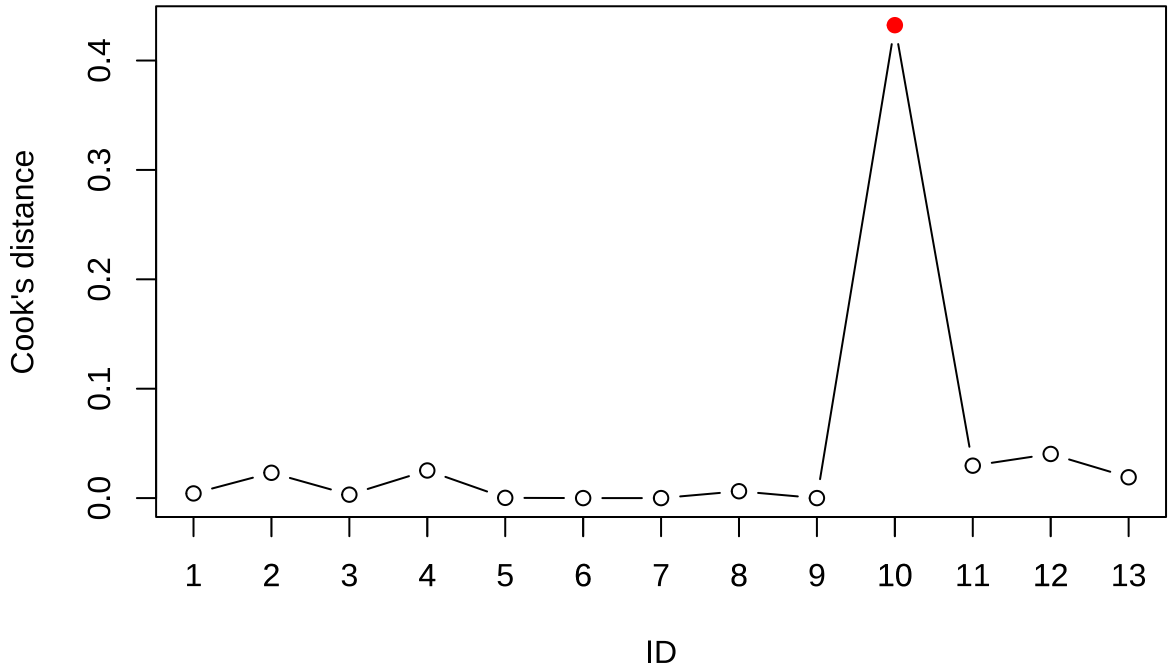

Note that study IDs follow alphabetical order of included studies and their specifications reported in the descriptive statistic's table

Comparing results

Table 1: Results of the three meta-analyses with different hypothesized correlations

Correlation	ES	I2	tau	CI	PI
r = .30	0.85	92.19	0.86	[0.35;1.35]	[-0.91;2.61]
r = .50	0.85	90.06	0.85	[0.35;1.35]	[-0.89;2.6]
r = .70	0.86	88.11	0.85	[0.36;1.36]	[-0.88;2.59]
